# Biological noise is a key determinant of the reproducibility and adaptability of cardiac pacemaking and EC coupling

**DOI:** 10.1085/jgp.202012613

**Published:** 2022-04-28

**Authors:** Laura Guarina, Ariana Neelufar Moghbel, Mohammad S. Pourhosseinzadeh, Robert H. Cudmore, Daisuke Sato, Colleen E. Clancy, Luis Fernando Santana

**Affiliations:** 1 Department of Physiology and Membrane Biology, University of California Davis School of Medicine, Davis, CA; 2 Department of Pharmacology, University of California Davis School of Medicine, Davis, CA

## Abstract

Each heartbeat begins with the generation of an action potential in pacemaking cells in the sinoatrial node. This signal triggers contraction of cardiac muscle through a process termed excitation–contraction (EC) coupling. EC coupling is initiated in dyadic structures of cardiac myocytes, where ryanodine receptors in the junctional sarcoplasmic reticulum come into close apposition with clusters of Ca_V_1.2 channels in invaginations of the sarcolemma. Cooperative activation of Ca_V_1.2 channels within these clusters causes a local increase in intracellular Ca^2+^ that activates the juxtaposed ryanodine receptors. A salient feature of healthy cardiac function is the reliable and precise beat-to-beat pacemaking and amplitude of Ca^2+^ transients during EC coupling. In this review, we discuss recent discoveries suggesting that the exquisite reproducibility of this system emerges, paradoxically, from high variability at subcellular, cellular, and network levels. This variability is attributable to stochastic fluctuations in ion channel trafficking, clustering, and gating, as well as dyadic structure, which increase intracellular Ca^2+^ variance during EC coupling. Although the effects of these large, local fluctuations in function and organization are sometimes negligible at the macroscopic level owing to spatial–temporal summation within and across cells in the tissue, recent work suggests that the “noisiness” of these intracellular Ca^2+^ events may either enhance or counterintuitively reduce variability in a context-dependent manner. Indeed, these noisy events may represent distinct regulatory features in the tuning of cardiac contractility. Collectively, these observations support the importance of incorporating experimentally determined values of Ca^2+^ variance in all EC coupling models. The high reproducibility of cardiac contraction is a paradoxical outcome of high Ca^2+^ signaling variability at subcellular, cellular, and network levels caused by stochastic fluctuations in multiple processes in time and space. This underlying stochasticity, which counterintuitively manifests as reliable, consistent Ca^2+^ transients during EC coupling, also allows for rapid changes in cardiac rhythmicity and contractility in health and disease.

## Teaser

Guarina et al. discuss recent findings suggesting that the high reproducibility of cardiac contraction emerges from high Ca^2+^ signaling variability at multiple levels due to stochastic fluctuations in multiple processes in time and space, but manifests as reliable Ca^2+^ transients during EC coupling.

## Introduction

The cardiac cycle beings with production of an action potential by pacemaking cells in the sinoatrial (SA) node. These action potentials, which exhibit a characteristic waveform reflecting the rapid, transient depolarization of the membrane and subsequent repolarization and afterhyperpolarization, are generated by clusters of pacemaker cells and propagate via gap junctions to surrounding atrial myocytes (Bleeker et al., 1980; James et al., 1966). The atrioventricular node serves as the point of entry for action potentials generated by depolarization of right and left atria, directing these electrical signals to septal Purkinje fibers, which rapidly propagate this electrical signal to right and left ventricles.

The process that links an action potential to cardiac muscle contraction is called excitation–contraction (EC) coupling, and the functional unit of EC coupling is called the “couplon” (Stern et al., 1997; Stern et al., 2013). A couplon is formed by clusters of L-type Ca^2+^ (Ca_V_1.2) channels in the sarcolemma and ryanodine receptors (RYRs) expressed in the juxtaposed junctional sarcoplasmic reticulum (jSR). In ventricular myocytes, the majority of couplons are formed along invaginations of the sarcolemma termed transverse tubules (T-tubules). In atrial myocytes, which lack a highly developed T-tubular system, couplons are primarily formed in the surface sarcolemma.

During EC coupling, membrane depolarization opens Ca_V_1.2 channels in the sarcolemma of atrial and ventricular myocytes. This allows a small amount of Ca^2+^ to enter the cytosolic nanodomain that separates the sarcolemma and jSR, increasing the Ca^2+^ concentration in this intracellular compartment ([Ca^2+^]_i_). This increase in [Ca^2+^]_i_ is sufficient to activate RYRs in the jSR via a Ca^2+^-induced Ca^2+^-release (CICR) mechanism (Fabiato, 1983) and is the initiating event in the EC coupling process. Activation of a small cluster of RYRs induces Ca^2+^-release events termed “Ca^2+^ sparks” (Cheng et al., 1993) that cause local [Ca^2+^]_i_ elevations. The synchronous activation of multiple Ca^2+^ sparks by Ca_V_1.2 channels throughout the myocyte summate to produce a transient global rise in [Ca^2+^]_i_ (hereafter referred to as [Ca^2+^]_i_ transients) that initiates myocardial contraction (Cannell et al., 1994, 1995; López-López et al., 1995). The coupling strength between Ca_V_1.2 channels and RYRs is critically dependent on the proximity and stability of the sarcolemmal–SR junction (Cannell and Soeller, 1997; Gomez et al., 1997; Soeller and Cannell, 1997).

The activation of couplons at sarcolemmal–jSR dyadic structures reflects Ca_V_1.2 and RYR channel gating, both of which are probabilistic processes (Cannell and Soeller, 1997; Soeller and Cannell, 1997; Stern et al., 1997, 2013). In couplons, information is therefore encoded by local Ca_V_1.2 channel–mediated Ca^2+^-influx events that are transmitted to RYRs, which ultimately decode this signal to produce Ca^2+^ sparks. These intrinsically probabilistic events propagate and produce fluctuations in local Ca^2+^ fluxes (i.e., signal) but also reflect stochastic behavior—that is, “noise”—of the couplon itself.

Ca^2+^ and electrical signals resulting from the opening of a single channel often exhibit relatively large event-to-event fluctuations due to variations in the open time of the channel. For an ion channel with a single open state, the probability of closing per unit time is constant; thus, the channel’s stochastic lifetimes are exponentially distributed. Accordingly, the SD of their lifetimes is equal to the mean, resulting in a coefficient of variation (COV = SD/mean) = 1.

In contrast, under control steady-state conditions, the COV of action potential–evoked transient increases in [Ca^2+^]_i_, reflecting the combined effects of Ca_V_1.2-mediated Ca^2+^ influx and synchronous RYR-mediated SR Ca^2+^ release, is ∼0.12 in adult mouse ventricular myocytes and ∼0.18 in neonatal rat ventricular myocytes (Vega et al., 2011), beat-to-beat fluctuations that are 5–10 times smaller than those of other signals produced by single molecules. Thus, under steady-state conditions, the reproducibility of the whole-cell [Ca^2+^]_i_ transient and contraction, at least in these cells, is very high compared with what would be expected for any stochastic single-molecule response.

Until recently, the general consensus was that the reliable consistency of the [Ca^2+^]_i_ transient in ventricular myocytes was likely produced by the activation of Ca^2+^ release from a permanently static SR structure that formed tight, functionally stable couplons throughout the myocyte. The stability of [Ca^2+^]_i_ transients, according to this thinking, was the result of the stochastic activation of a temporally and spatially averaged number of SR Ca^2+^-release units. However, recent work suggests that the SR and jSR (Drum et al., 2020; Vega et al., 2011) as well as Ca_V_1.2 (Ghosh et al., 2018; Sato et al., 2019) and RYR2 clusters (Asghari et al., 2020; Hiess et al., 2018)—and even T-tubules (Song et al., 2006; Wagner et al., 2012)—are dynamic structures. Furthermore, changes in SR Ca^2+^ load, SR Ca^2+^ flux, and rate of Ca_V_1.2 channel inactivation have now been shown to change in a beat-to-beat fashion to tune cardiac contractility (Eisner et al., 2017; Smith and Eisner, 2019).

In this review, we provide an analysis of recent discoveries regarding the mechanisms underlying cardiac pacemaking activity as well as couplon formation and plasticity and how they impact EC coupling and cardiac performance. Using amalgamated data, we propose a new framework that accounts for the remarkable cardiac reliability and reproducibility by incorporating network-, cellular-, and subcellular-event sources of noise. An important feature of this framework is that cellular and molecular events that contribute to noise can paradoxically enhance cardiac rhythmicity and variability of EC coupling, depending on their timing and magnitude; thus, these noise-generating Ca^2+^ events become regulatory “nodes” that tune cardiac contractility.

## Statistical analyses of random events

A question that nearly every investigator with even a passing interest in cardiac EC coupling encounters at one time or another, regardless of background or particular research focus, is how much of their data reflect the operation of stochastic processes. For example, a cell biologist may ask whether membrane proteins form clusters and colocalize to form multiprotein signaling complexes through a stochastic process or an active targeting mechanism. Are cytoskeletal structures such as microtubules randomly anchored along the sarcolemma of ventricular myocytes? Do jSRs form dyads at random locations on T-tubules? Or are these various molecular structures directed to preferred locations? Likewise, is activation of RYR2 or Ca_V_1.2 channel gating stochastic or coupled? To answer such questions, researchers need to undertake statistical analysis to test the null hypothesis that the data describe a stochastic process.

Before proceeding, some definitions are in order. A stochastic process is one for which the probability of making a particular observation is given by a random variable. Note that, although the two terms are often used synonymously, stochasticity and randomness are not the same. While stochasticity refers to a modeling approach for a system, randomness refers to individual probabilistic, nondeterministic events that fluctuate and contribute to variance (i.e., noise). The binomial distribution and related Poisson, exponential, and Gaussian distributions, which provide a theoretical framework for statistical analyses of stochastic processes, are defined below with representative examples of their applications.

The “binomial distribution” is a discrete probability distribution of the number of successes in a sequence of experiments in which the following four conditions are met: first, the number of observations is fixed; second, each observation is independent; third, each observation represents one of two outcomes, success or failure; and fourth, the probability of success is the same for each trial. The binomial distribution is described by the following equation:

$$P\left(x\right)=\left[N!/\left(N-x\right)!x!\right]\times P^{x}\times {\left(q\right)}^{N-x}\text{,}$$ (1)

where *P* is the probability of success, *N* is the number of successes, and *q* is the probability of failure (i.e., 1 − *P*). The binomial distribution has been used to analyze unitary events such as single-channel currents (Sakmann and Neher, 2009) and elementary Ca^2+^ signals (TRPV4 sparklets) produced by Ca^2+^ influx via single transient receptor potential vanilloid 4 (TRPV4) channels in endothelial cells (Sonkusare et al., 2014).

The Poisson distribution, another discrete probability distribution, expresses the probability of a given number of events occurring in a fixed interval of time or space if these events occur with a known constant mean rate independently of the time since the last event. If it has a probability mass function, that is, a function that gives the probability that a discrete random variable is exactly equal to some value, a Poisson distribution is given by the following:

P(x)=e−λ×λxx!, (2)

where *P* is probability of success, *e* is 2.718, λ is the mean, and *x* is the number of trials. In a process that follows a Poisson distribution, the mean (λ) and variance are equal. Indeed, the Poisson distribution is a limiting case of a binomial distribution where the number of trials is very large (e.g., *N* > 100) and the probability of success is small (e.g., *P* < 0.1). These conditions are met by evoked Ca^2+^ sparks in ventricular myocytes. Cannell et al. (1995) used Poisson statistics to test the null hypothesis that Ca_V_1.2 channel–activated Ca^2+^ sparks occur randomly throughout ventricular myocytes. They found that the probability density function (i.e., the probabilities of a random variable for a range of values) of evoked Ca^2+^ sparks was generated by quantifying the number of occurrences (i.e., *P* in Eq. 2) of a specific number of Ca^2+^ sparks per 1.8 μm (i.e., *x* in Eq. 2) from all the images. The resulting histogram was well fitted to a Poisson function with a mean (i.e., λ in Eq. 2) of 2.2 Ca^2+^ sparks per 1.8 μm. On the basis of this analysis, they concluded that Ca^2+^ spark activation by Ca_V_1.2 channels was stochastic.

The exponential distribution is the probability density function for a Poisson process in which events occur continuously and independently but at a constant rate. That is, if the number of events per unit time follows a Poisson distribution, then the amount of time between events follows an exponential distribution. An example is the amount of charge flowing through an ion channel when it opens. After opening, a channel with a single open state has a constant probability of closing per unit time (Hille, 2001). The stochastic open times of such a channel are exponentially distributed, and the mean and SD of the lifetimes are equal. This results in a COV = 1.

A Gaussian distribution is a type of continuous (as opposed to discrete, as in binomial and Poisson distributions) probability distribution of normally distributed stochastic processes. Indeed, the Gaussian distribution can be considered as another special case of the binomial, when the number of trials is sufficiently large. For this reason, the Gaussian distribution applies to a large number of variables.

Two relevant implementations of this analysis, one by Wang et al. (2001) and the other by Navedo et al. (2005), were performed to analyze the amplitudes of Ca^2+^ sparklets—local elevations in [Ca^2+^]_i_ produced by the opening of single Ca_V_1.2 channels or a small cluster of channels—in cardiac and smooth muscle cells. The analysis begins by generating histograms of Ca^2+^ sparklet event amplitudes from [Ca^2+^]_i_ records. Their Ca^2+^ sparklet amplitude histograms showed multiple peaks that could be fitted to the following multicomponent Gaussian function:

$$P=\sum_{j=1}^{n}a_{j}\times \exp\left[-\frac{{\left(\Delta \left[Ca^{2+}\right]i-jq\right)}^{2}}{2jb}\right]\text{,}$$ (3)

where *a* and *b* are constants, and Δ[Ca^2+^]_i_ and *q* are the change in intracellular Ca^2+^ concentration and quantal unit of Ca^2+^ influx, respectively. This analysis allowed Wang et al. (2001) and Navedo et al. (2005) to determine the quantal unit of Ca^2+^ sparklet amplitude and conclude that large-amplitude events resulted from the synchronous activation of quantal Ca^2+^ sparklet events.

## Implications of noise in biological systems

The behavior of a dynamic biological system such as a cardiac myocyte is subject to both intrinsic and extrinsic noise (i.e., stochasticity). Accordingly, under steady-state conditions, pacemaking and EC coupling are stochastic processes, and their properties may be represented statistically as probability distributions that are weighted toward achieving a mean level of action potential periodicity as well as Ca^2+^ influx and SR Ca^2+^ release, but cannot be definitively predicted. Instead, the state of the system may fluctuate around a mean value determined by the initial conditions, unless the system is multistable. This fluctuation, or deviation from the target state, is the result of the underlying stochasticity of all the processes involved.

**Figure 1.**
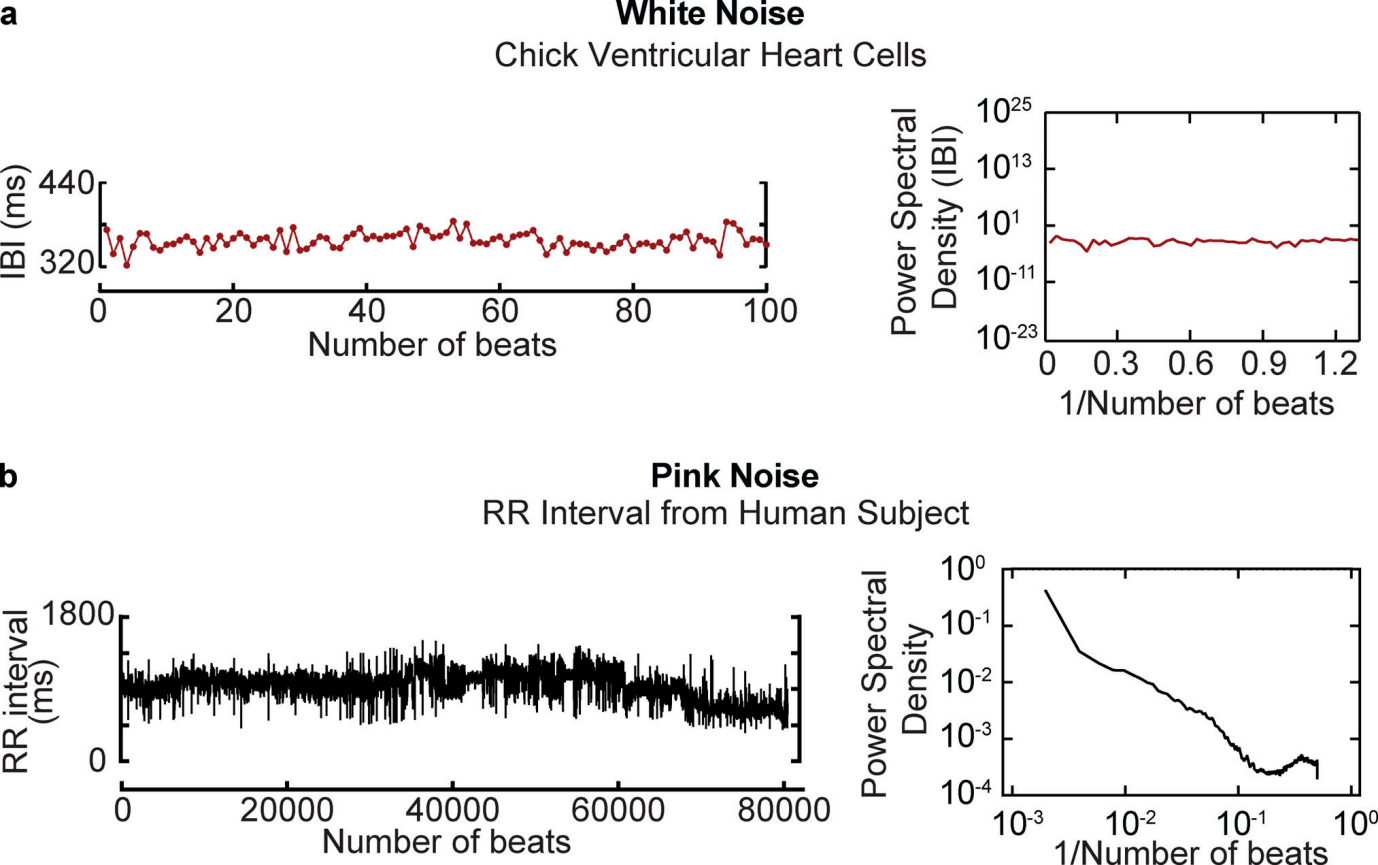
**Time and frequency profiles of Gaussian and pink noise. (a)** Example of white noise. Data in this panel were reproduced from Krogh-Madsen et al. (2017) and show the time course of interburst intervals (left column) recorded from a chick ventricular cardiomyocyte and corresponding power spectral density (right column). **(b)** Example of pink noise. Time course of RR interval from a 53-yr-old man (left column) obtained from Irurzun et al. (2021). The power spectral distribution of this trace is shown in the right column.

Before detailing the mechanisms by which noise impacts pacemaking and EC coupling, we briefly consider two biologically relevant types of noise: white noise and pink noise (Fig. 1). The characterization of white and pink noise—indeed, the characterization of noise in general—is usually framed in terms of frequency. In Fig. 1 a, a record of the time course of interburst intervals and their power spectral distribution from a chick cardiomyocyte are shown (Krogh-Madsen et al., 2017). Note that the power spectral distribution in this example has equal intensities at all frequencies, producing a constant power spectral density that is typical of white noise (Fig. 1 a). Pink noise has a power spectral density that is inversely proportional to frequency (i.e., 1/*f*). An example of 1/*f* noise is fluctuation of the heartbeat period (Garavaglia et al., 2021; Irurzun et al., 2021; Kobayashi and Musha, 1982; Fig. 1 b).

White noise can be further classified as either uniform or Gaussian. The spectral power in Gaussian white noise is uniformly distributed across all frequencies and is normally distributed in the time domain. Gaussian noise is one of the most common descriptors of fluctuations in biological systems and is therefore implemented in simulations of ionic currents and EC coupling (e.g., Aghasafari et al., 2021; Sato et al., 2009; Krogh-Madsen et al., 2017; Guevara and Lewis, 1995). However, as discussed in further detail below, there is a paucity of analyses of [Ca^2+^]_i_ and electrical noise during EC coupling and therefore of computational models that incorporate experimentally determined noise; without inclusion of these data, the predictions of these models are altered.

**Figure 2.**
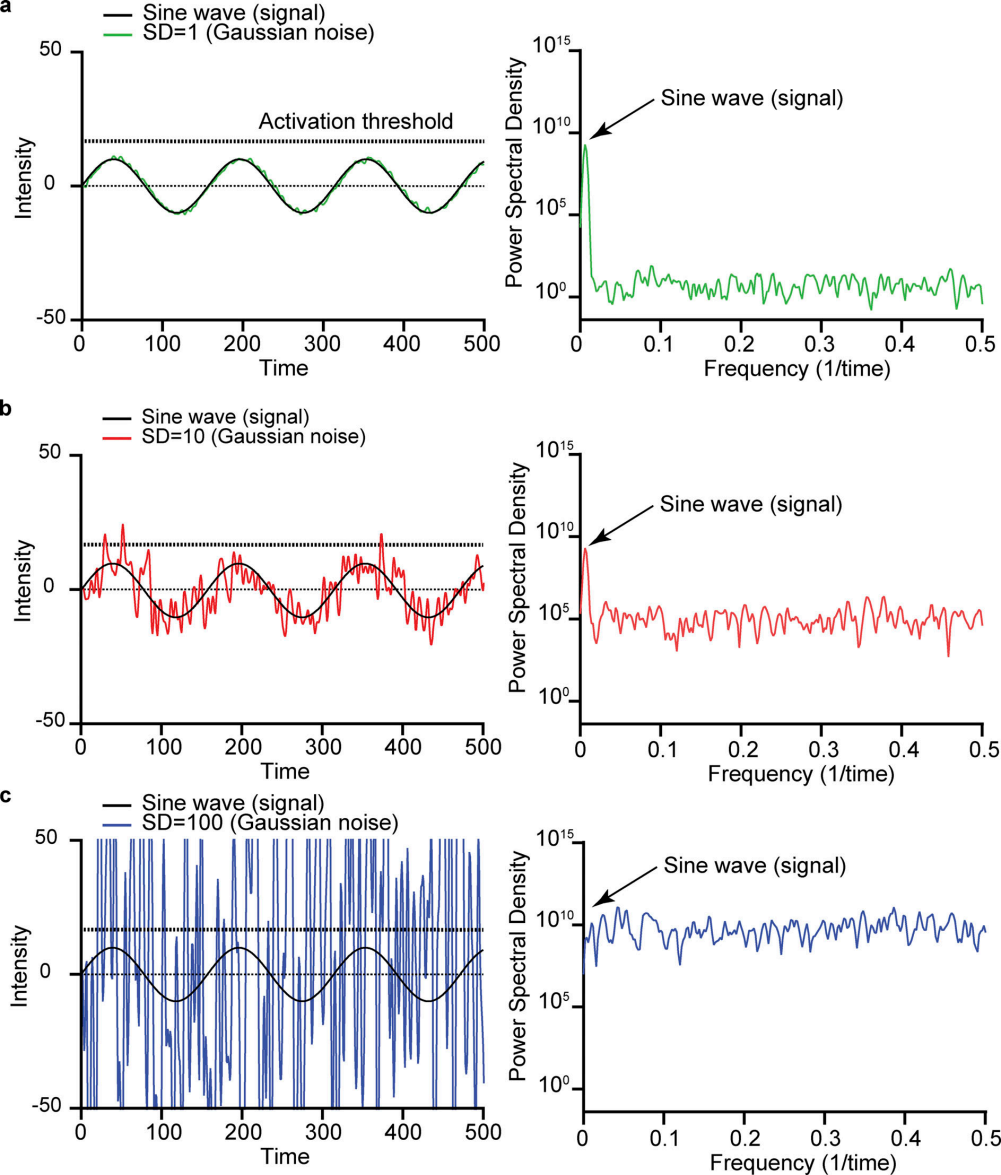
**Increases in the amplitude of Gaussian noise diminish signal detection and discrimination.** **(a–c)** In this figure, an arbitrary sinusoidal signal (black trace, left column) is superimposed on the composite of the original signal added to Gaussian white noise of increasing variance (colored traces). Summation of Gaussian white noise with a mean of 0 and SD of 1 (a), 10 (b), and 100 (c) to an arbitrary sinusoidal signal (*y* = 10 sin [*x*/2]; black line). The power spectral density plot of the composite signal and noise values is shown in the right column.

Fig. 2 shows the time course of a sinusoidal wave (0.006 Hz) with an arbitrary mean intensity of 0 arbitrary units with increasing levels of white Gaussian noise and a detection threshold set at 12.5. We provide three SDs (Fig. 2, a–c, left column) corresponding to three COV scenarios. The plots in the right column of each panel show the power spectral density of each signal and noise combination. A key aspect of these power spectral density distributions is that they show the amplitude of the sine wave relative to the Gaussian noise. Note that as the noise amplitude is increased, the power spectral density distribution of the noise eventually surpasses that of the sine wave signal. This has important implications. For example, consider a system in which activation under each of the variance scenarios requires a time-dependent signal with a peak amplitude of 12.5 arbitrary units. Such a signal would not be detected when the SD is 1, as none of the noise fluctuations would reach the detection threshold. Note that with an SD of 10, multiple noise fluctuations events cross the detection threshold over the 100-s simulation. However, with an SD of 100, whether the system reaches a threshold is determined solely by the noise.

These simple scenarios allow us to make two critical points. The first is that noise fluctuations can aid a periodic signal in reaching a detection threshold. The second is that, when noise levels are high, they can blur the underlying signal and may lead to random, high-frequency detection and loss of periodicity. As discussed below, these two points have significant implications for cardiac rhythmicity and EC coupling in health and disease.

## Noise in cardiac pacemaking: entrainment versus stochastic resonance

We begin our discussion of noise in cardiac function with pacemaking by the SA node, where, in a normal heart, an action potential initiates each cardiac cycle. Luigi Galvani, working in the late 1700s, was the first to propose the concept of an electrical pacemaker in the heart based on his discovery that contraction of a frog heart could be induced by injecting an electrical current (Boyett and Dobrzynski, 2007). Since that seminal discovery more than 200 years ago, the mechanisms underlying cardiac pacemaking have been actively investigated. These efforts have accelerated over the last four decades thanks to major advances in cellular electrophysiology, molecular biology, and optical microscopy, which have led to the formulation of a model for spontaneous action potential production by SA myocytes (Bogdanov et al., 2001a; Brown and Difrancesco, 1980; Cho et al., 2003; Choudhury et al., 2015; DiFrancesco, 1991; Lakatta et al., 2010; Tsutsui et al., 2018). Briefly, the action potential of SA node myocytes is initiated by the progressive depolarization of these cells from their maximum diastolic membrane potential (approximately −60 to −50 mV). This depolarization is driven, at least in part, by the activation of hyperpolarization-activated cyclic nucleotide-gated channels 2 and 4 (HCN2 and HCN4) currents (Baruscotti et al., 2005; Brown and Difrancesco, 1980; DiFrancesco and Tortora, 1991). The concomitantly evoked Ca^2+^ sparks activate inward Na^+^/Ca^2+^ exchanger currents, which further contribute to diastolic depolarization (Bogdanov et al., 2001b; Bychkov et al., 2020; Huser et al., 2000; Ju and Allen, 1998). As the cell membrane becomes more depolarized, voltage-gated T-type Ca_V_3.1 channels (Mangoni et al., 2006), L-type Ca_V_1.3 (Christel et al., 2012; Mangoni et al., 2003), and shortly thereafter, L-type Ca_V_1.2 channels (Christel et al., 2012; Kodama et al., 1997) are activated, increasing the rate of depolarization and leading to a fully developed action potential. This process triggers a transient global increase in [Ca^2+^]_i_. The repolarizing phase of the SA node action potential starts with the inactivation of Ca^2+^ currents, followed by the opening of voltage-gated K^+^ currents and Ca^2+^-sensitive K^+^ channels (Haron-Khun et al., 2017; Weisbrod et al., 2016). As SA node cells reach hyperpolarized potentials, HCN channels open to depolarize the cell and allow the cycle to be repeated.

**Figure 3.**
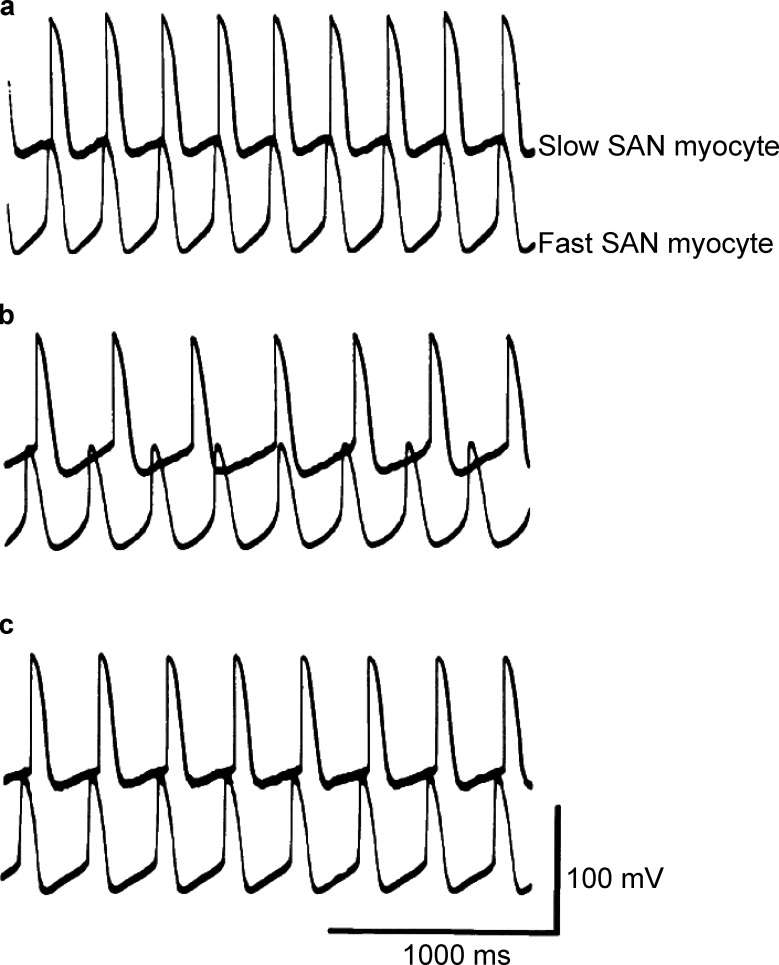
**Entrainment of SA node (SAN) myocytes. (a–c)** Simultaneous records of spontaneous action potentials from slow- and fast-firing myocytes in an intact SA node under control conditions (a), during high sucrose (b), and after returning to control conditions (c). Data are from Jalife, 1984.

For decades, the generally accepted model of pacemaking activity was based on an entrainment mechanism (Anumonwo et al., 1991; Hennis et al., 2021; Jalife, 1984)—the process by which independent, self-sustaining rhythmic elements that share some form of oscillatory activity interact with each other so as to fire in unison. To illustrate how entrainment may develop in the SA node, in Fig. 3, we show simultaneously recorded action potentials from fast- and slow-firing myocytes from an intact rabbit SA node in a sucrose gap preparation (see Jalife, 1984). In this experimental setup, the SA node is placed in a chamber with three compartments, and electrical recordings are made from cells in different compartments. The three compartments allow different solutions to be applied to different sections of the intact SA node. Under control conditions, with all sections of the node exposed to a physiological saline solution, the fast- and slow-firing myocytes fire action potential synchronously, with the faster myocyte setting the pace (Fig. 3 a). Superfusion of a sucrose solution with low ionic concentration onto the central compartment of the preparation decouples the fast and slow pacing cells (Fig. 3 b), likely because of decreased electrical coupling. Reintroduction of the control saline solution into the central compartment restores synchronization of action potentials in fast and slow SA node myocytes. These data suggest a model in which phasic entrainment occurs when two cells firing action potentials at two slightly different rates or phases are synchronized into one distinct phase and pace of firing. When the action potential of one of the cells arrives as the other cell repolarizes, the timing of the next action potential is delayed (i.e., phase delay). However, if one of the cells fires an action potential when the other cell is undergoing diastolic depolarization, it could trigger an earlier action potential (i.e., phase advance).

More recent work from the Fenske lab (Fenske et al., 2020; Hennis et al., 2021) has suggested an additional mechanism for SA node entrainment: tonic. Tonic entrainment can occur when the maximum diastolic potential of a SA myocyte becomes transiently hyperpolarized relative to that of its neighbors. Under these conditions, transiently hyperpolarized myocytes may enter a nonfiring mode and becoming a current sink. Electrically coupled cells would then become hyperpolarized, and thus their firing rates would decrease. The opposite would happen if the maximum diastolic potential were to become more depolarized.

In the entrainment model, the dominant pacemaking site in the SA node need not necessarily be static (Boyett et al., 2000; Kodama et al., 1997; Yamamoto et al., 1998). Instead, it has been proposed to dynamically shift within the node in response to physiological stimuli such as activation of the autonomic nervous system (Brennan et al., 2020).

**Figure 4.**
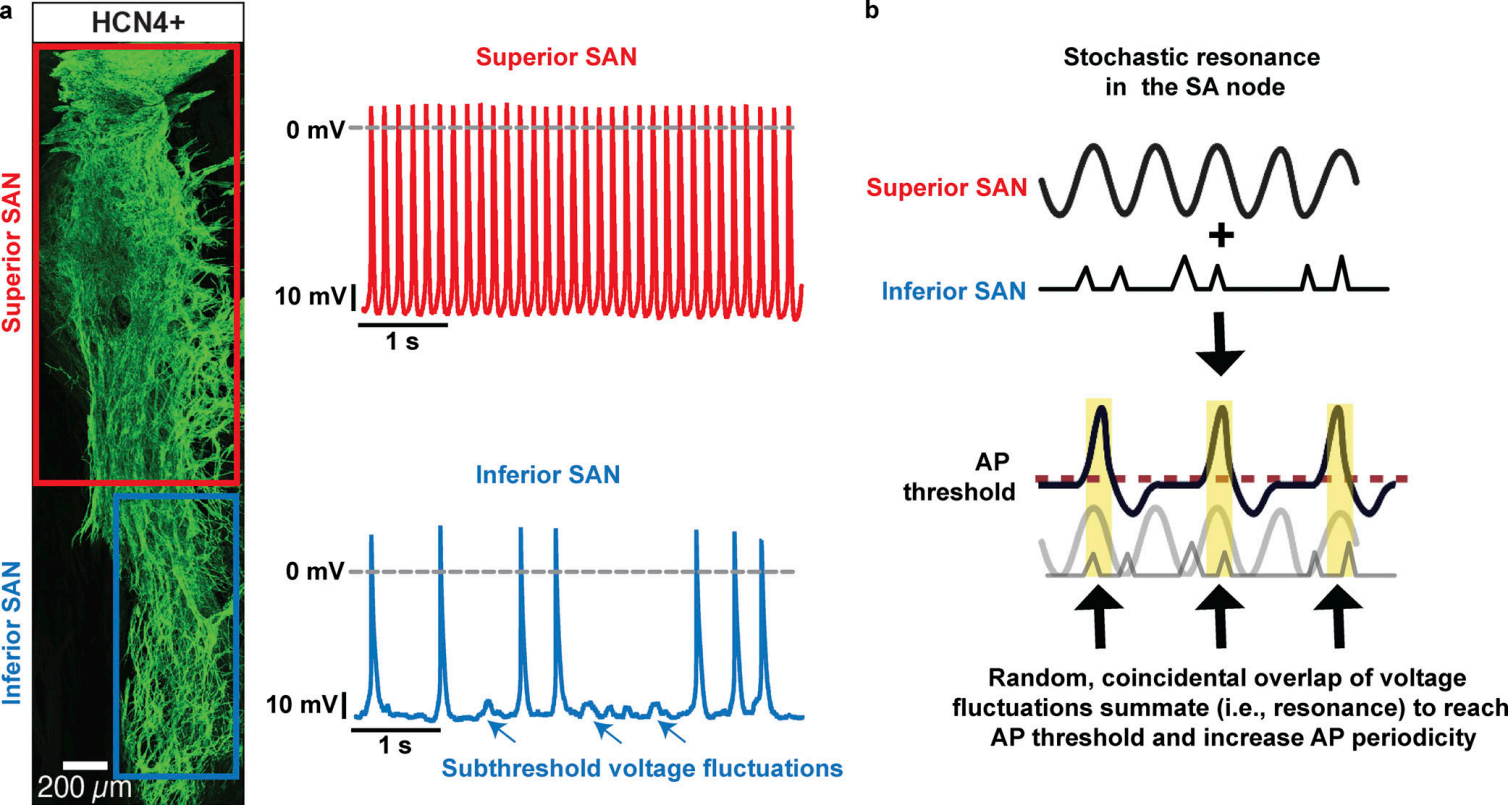
**Depiction of the stochastic resonance model of cardiac pacemaking. (a)** Left: Confocal image of HCN4^+^ myocytes in a mouse SA node (SAN). The superior section of the node is densely populated by HCN4-expressing myocytes. The inferior SAN has a lower myocyte density. Right: Membrane potential records from representative superior and inferior SAN myocytes. Superior SAN myocytes (top) fire action potentials at a higher frequency than inferior myocytes (bottom). The majority of inferior SAN myocytes fire random action potentials or subthreshold voltage fluctuations (arrows). **(b)** Stochastic resonance model, in which the superior node functions as a periodic oscillator and the inferior as a noise generator. Subthreshold superior oscillations, when they occur with simultaneous random noise input from the inferior, could exhibit stochastic resonance and produce more robust spiking. Data are from Grangier et al., 2021.

A key feature of the SA node is its high anatomic and functional heterogeneity (Boyett et al., 2000; Bychkov et al., 2020; Kim et al., 2018; Monfredi et al., 2018; Oren and Clancy, 2010). Grainger et al. (2021) showed that, in male mice, myocyte density in the superior region of the node is high (Fig. 4 a). Concomitantly, the superior section of the SA node is populated by myocytes with a higher intrinsic action potential firing rate than inferior myocytes. Indeed, a relatively large fraction of inferior SA node myocytes produce random subthreshold voltage fluctuations and/or action potentials. Based on these findings, an alternative model was recently proposed in which pacemaking activity by the SA node is produced by a stochastic resonance mechanism (Clancy and Santana, 2020; Grainger et al., 2021; Fig. 4 b). In general terms, stochastic resonance, a phenomenon in which a weak signal is amplified by adding noise to it, occurs when noise has a positive role in a signal-processing context. The system resonates with the frequencies in the noise that correspond to the system’s natural frequencies, thus amplifying the oscillations. The data in Fig. 2, a–c, provide an example of how this could happen. The periodicity with which a system reaches the activation threshold is low at low noise levels but increases as the level of noise is increased, reaching maximum performance at the resonance point. At high levels, however, activation of the system is dominated by random noise, decreasing periodicity (i.e., performance).

In the stochastic resonance pacemaking model, SA myocytes could operate as a bistable system that switches from hyperpolarized potentials to a fully developed action potential. One important element of the model is that it incorporates the anatomic and functional heterogeneity of the firing modalities of cells, which combine to form a very organized heartbeat, even when cell-to-cell connectivity via gap junctions is weak owing to low expression of connexins (Anumonwo et al., 1992; Boyett et al., 2000, 2006). Most SA node myocytes in the inferior section of the node fire stochastic subthreshold voltage fluctuations or rare single action potentials. A fraction of the cells can fire action potentials in bursts (Fenske et al., 2020; Grangier et al., 2021). These signals do not lead to periodic pacemaking on their own. However, when coupled to more periodic voltage oscillators, such as superior SA node myocytes, random subthreshold voltage fluctuations and action potentials integrate (i.e., resonance effect) to increase the probability that superior SA node myocytes reach the action potential threshold. Thus, the noisy SA node myocytes likely increase the strength and periodicity of tonically firing SA node myocytes and hence their pacemaking activity. A key feature of this model is that inferior SA node myocytes do not fire action potentials at high frequencies for prolonged periods of time even if they are intrinsically capable of doing so because they do not have a sufficient blood supply to sustain their electrical activity.

**Figure 5.**
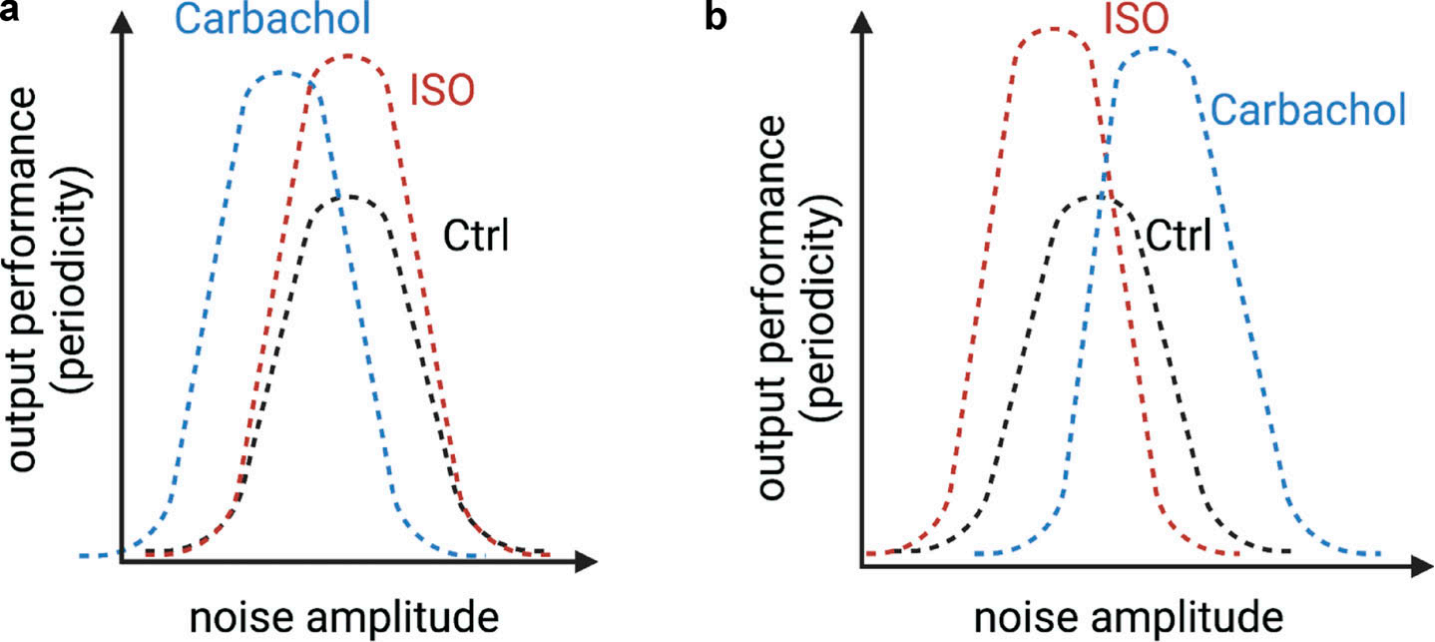
**Predicted noise–performance relationship for a stochastic resonance model of pacemaking activity.** Hypothetical plots of the relationship between noise and pacemaking periodicity under control conditions and during activation of sympathetic nervous system (SNS) and parasympathetic nervous system (PNS) signaling. **(a)** Plot assumes that sympathetic and parasympathetic stimulation only increase peak performance, leaving the response to the noise amplitude unchanged. **(b)** Plot assumes that sympathetic and parasympathetic stimulation shift the noise amplitude relationship toward a preference for lower values in the case of SNS activation and larger values for PNS activation, while increasing peak performance. ISO, isoproterenol.

Future studies should test entrainment and stochastic resonance models experimentally and in silico. A testable prediction of the entrainment model is that, over time, all cells within the SA node should discharge synchronously. This is not the case for the stochastic resonance model, where not all SA node myocytes would discharge at the same frequency. Furthermore, the stochastic resonance model predicts that SA performance (e.g., periodicity) would be low at low noise levels (Fig. 5). Consistent with this, Yaniv et al. (2014) found that decreasing the magnitude of HCN or SR Ca^2+^-induced random voltage fluctuations increased beat-to-beat variability. Periodicity would increase as the level of noise increased, reaching maximum performance at the resonance point. Increasing noise beyond this point would decrease SA node periodicity, as pacemaking activity would come to be dominated by random voltage noise.

Future research should also determine the relationship between performance (e.g., inter–action potential periodicity) and noise (e.g., amplitude of subthreshold voltage fluctuations) and how activation of sympathetic and parasympathetic signaling affect the response. Two potential outcomes of these stochastic resonance experiments are illustrated in Fig. 5 (a and b). Under control conditions, the stochastic resonance model predicts a bell-shaped noise–performance relationship. An interesting question would be whether activation of the autonomic nervous system alters this relationship. For example, one reasonable prediction is that sympathetic and parasympathetic stimulation would simply increase the magnitude of pacemaking periodicity by increasing cell synchronization without shifting the noise–performance relationship (Fig. 5 a). Alternatively, and perhaps more realistically, activation of the autonomic nervous system might increase the peak and shift the noise–performance relationship. For example, sympathetic stimulation might shift the noise–performance plot to the left as the cells become more hyperpolarized and require larger currents to reach the action potential threshold. By contrast, parasympathetic stimulation might shift the noise–performance relationship to the right as cells become more hyperpolarized (Fig. 4 b). Whether the amplitude of the noise–performance relationship increases during sympathetic and parasympathetic stimulation as SA node cells become more synchronized would also be interesting to investigate (Goldberger et al., 1994; He, 2020).

An additional, critically important experiment would involve elimination or attenuation of electrical signaling of the inferior section of the node (which is largely populated by cells that produce stochastic subthreshold voltage fluctuations) while recording action potential firing from the whole tissue. The stochastic resonance model predicts that doing so would lead to decreased pacemaking periodicity (i.e., inter–action potential variance).

Regardless of whether SA node pacemaking activity depends on entrainment or stochastic resonance, testing these models would provide important insights into mechanisms that lead to normal sinus rhythm.

## SR Ca^2+^ release is responsible for the majority of the [Ca^2+^]_i_ variance during ventricular EC coupling

**Figure 6.**
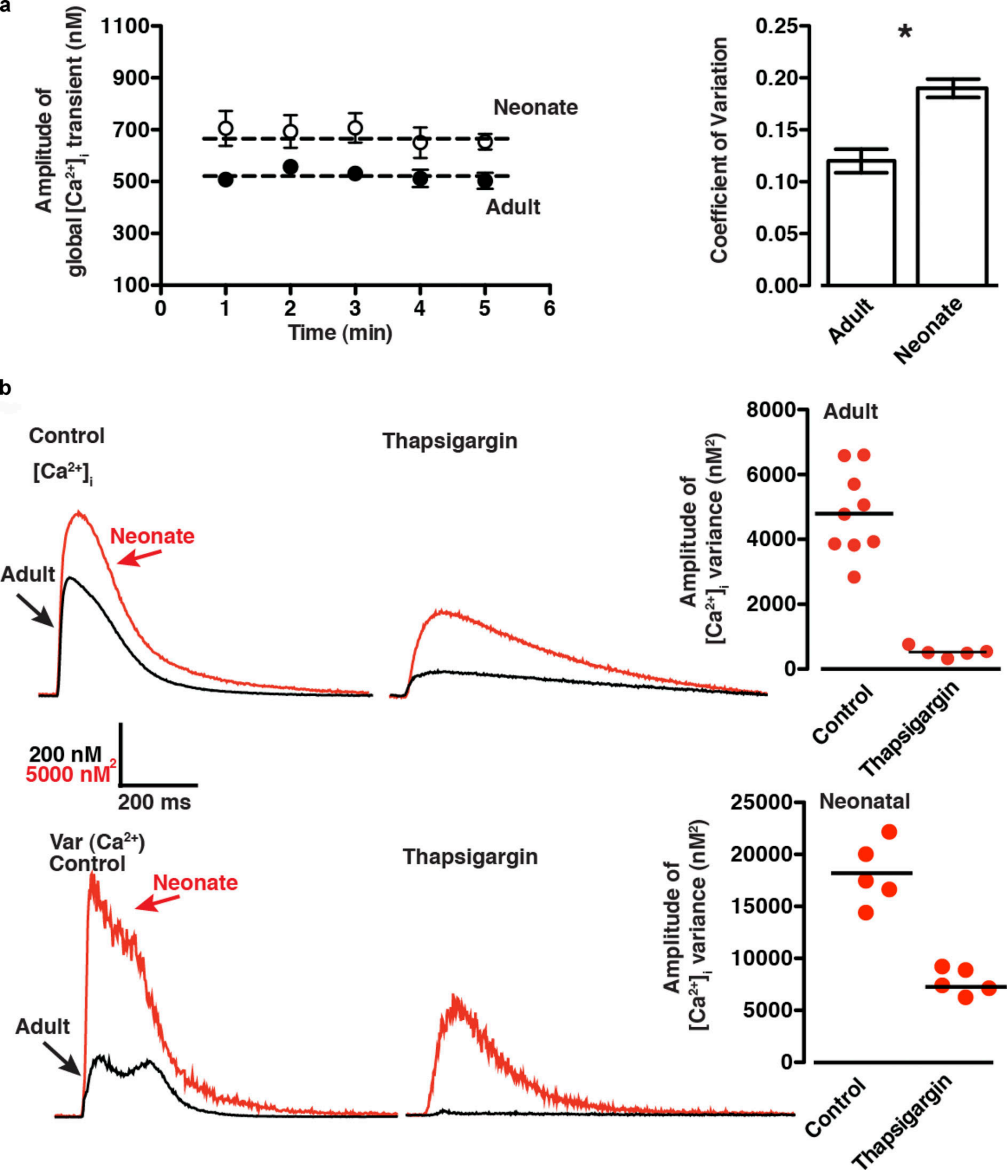
**SR Ca**^**2+**^ **release is the largest source of beat-to-beat [Ca**^**2+**^**]**_**i**_ **variability in adult and neonatal ventricular myocytes. (a)** Average peak amplitude of action potential–evoked (1-Hz) global [Ca^2+^]_i_ transients from adult and neonatal ventricular myocytes, measured at 1-min intervals for 5 min, and the corresponding COV among adult and neonatal myocyte populations. The dashed line represents peak [Ca^2+^]_i_ signal averaged over 5 min. * denotes statistical significance. **(b)** Representative averaged [Ca^2+^]_i_ transient and associated signal variance of adult and neonatal ventricular myocytes and the distribution of peak amplitude [Ca^2+^]_i_ variance (nM^2^) of adult ventricular myocytes in the presence and absence of the SERCA inhibitor thapsigargin (1 μM). Figure from Vega et al., 2011.

**Figure 7.**
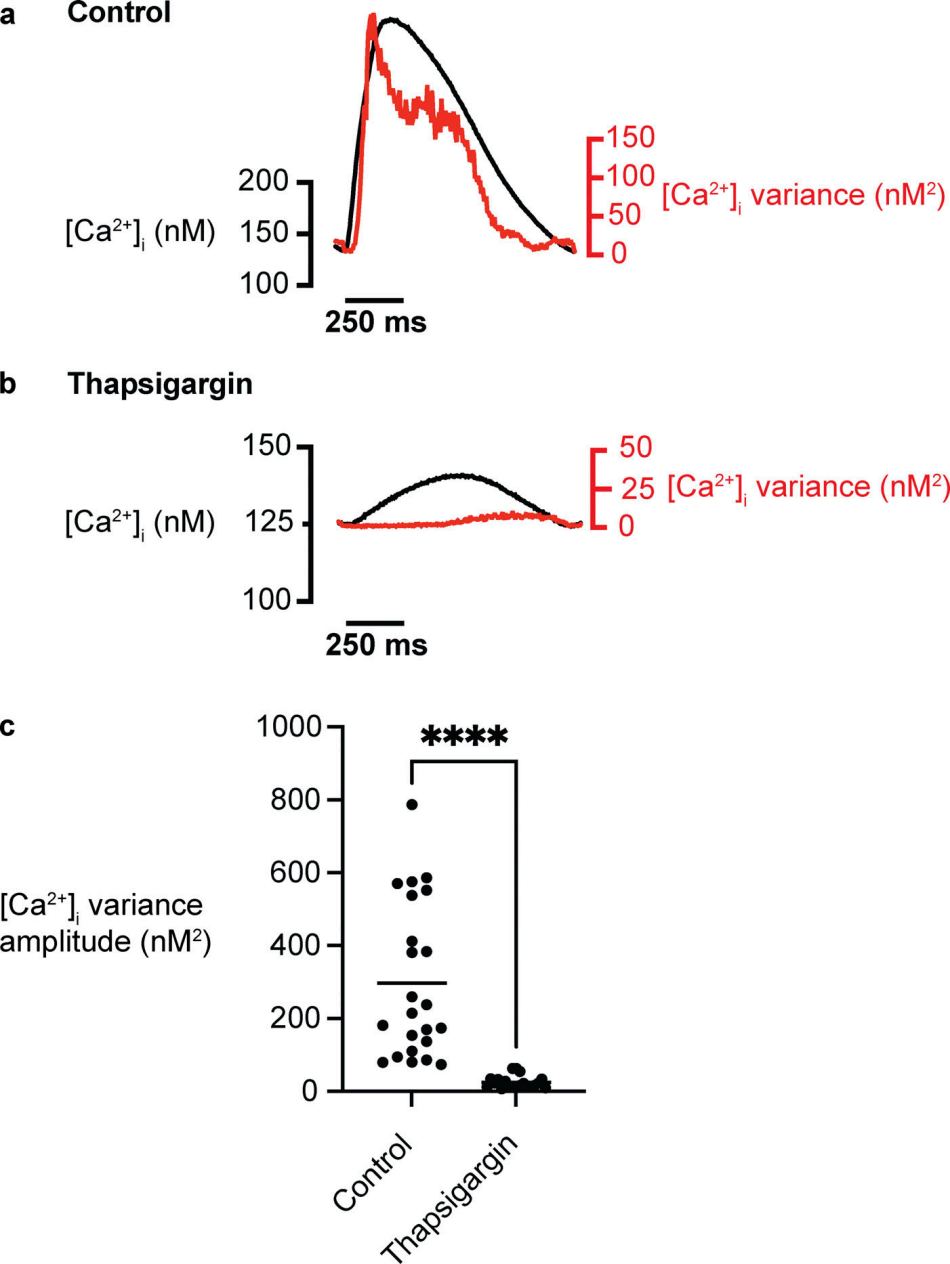
**SR Ca**^**2+**^ **release is the largest source of beat-to-beat [Ca**^**2+**^**]**_**i**_ **variability in adult rabbit ventricular myocytes. (a and b)** Representative averaged [Ca^2+^]_i_ transient and associated signal variance of adult rabbit ventricular myocytes and the distribution of peak amplitude [Ca^2+^]_i_ variance (nM^2^) of adult ventricular myocytes under control conditions (a) and in the presence of the SERCA inhibitor thapsigargin (1 μM; b). **(c)** Population data for the amplitude of [Ca^2+^]_i_ variance during EC coupling (control, *n* = 23 cells; thapsigargin, *n* = 20 cells; ****, P < 0.0001).

Live-cell imaging is a powerful tool for studying EC coupling noise at the single-cell level, as it has the high spatiotemporal resolution necessary to detect the dynamics of [Ca^2+^]_i_ at this level and sufficient throughput to gather the volume of data needed for these noise analyses. Using these approaches, Vega et al. (2011) imaged action potential–evoked [Ca^2+^]_i_ transients (1-Hz stimulation rate) in adult mouse ventricular myocytes and neonatal rat ventricular myocytes (≤3 d old) and determined the time course of beat-to-beat variations in [Ca^2+^]_i_ (Fig. 6). The authors made two interesting observations regarding [Ca^2+^]_i_ noise during EC coupling. First, the COV of the [Ca^2+^]_i_ transient peak amplitude over a 5-min period was 0.12 in neonatal rat ventricular myocytes and 0.18 in adult myocytes (Fig. 6 a). A similar experimental approach for determining [Ca^2+^]_i_ variance (σ^2^) during EC coupling in rabbit ventricular myocytes (2.5-Hz stimulation rate), implemented for this review (Fig. 7), yielded COVs for the [Ca^2+^]_i_ transient of 0.18 ± 0.2 at 2.5 Hz. These data suggest that beat-to-beat reproducibility is very high in rat neonatal cardiomyocytes as well as adult mouse and rabbit ventricular myocytes, at least under the experimental conditions used. The second important observation was that, by generating signal-averaged [Ca^2+^]_i_ records and their associated variance before and after eliminating SR Ca^2+^ release using the SR/ER ATPase (SERCA) inhibitor thapsigargin, it was possible to determine the time course of [Ca^2+^]_i_ variance during the action potential and the contribution of SR Ca^2+^ release to it (Figs. 6 b and 7 a).

In adult mouse and rabbit ventricular myocytes, in which ∼80–90% of the Ca^2+^ that produces the [Ca^2+^]_i_ transient comes from the SR (Li et al., 1998), [Ca^2+^]_i_ variance is highest at or near the peak of the [Ca^2+^]_i_ transient but decays with time. Interestingly, application of thapsigargin decreased [Ca^2+^]_i_ variance, on average, nearly 10-fold, suggesting that a larger portion of the action potential–associated [Ca^2+^]_i_ variance is linked to SR Ca^2+^ release in mouse and rabbit ventricular myocytes under these experimental conditions (Fig. 6 b; and Fig. 7, b and c).

An important consideration in this analysis is that an increase in the emission light intensity could translate into an increase in the shot noise associated with the photomultiplier tube (PMT) detector of the confocal microscope used in these experiments. To determine the magnitude of these fluctuations, we measured the variance of the fluorescence signals from two solutions with identical fluo-4 concentrations (100 μM) but with 100 or 600 nM free Ca^2+^. The solutions were placed on the stage of our microscope and imaged using the same lens, laser intensity, and PMT gain used during Ca^2+^ imaging experiments involving myocytes. The goal was to measure the variance of the Ca^2+^ signal over a similar range of fluorescence intensity changes (approximately threefold) during an action potential. The variance was 3.9 ± 0.3 and 11.3 ± 2.3 nM^2^ at 100 and 600 nM free Ca^2+^, respectively. This shows that, at least in our system, the nonbiological component (e.g., PMT shot noise) of the variance at the higher [Ca^2+^]_i_ levels and fluorescence intensities in the cell-free system was ∼27-fold smaller than the variance associated with the peak of the [Ca^2+^]_i_ transient of a rabbit ventricular myocyte (i.e., 297.4 ± 44.3 nM^2^) during the action potential. Thus, although nonbiological noise increases as fluorescence intensities increase, under our experimental conditions, it is a relatively small fraction of the measured [Ca^2+^]_i_ variance in a cell.

Ca^2+^ release from the SR plays a less prominent role in the [Ca^2+^]_i_ transient of rat neonatal myocytes (∼60–70%) compared with rat adult myocytes (∼90%; Kirby et al., 1992; Korhonen et al., 2009). As is the case in adult mouse ventricular myocytes, [Ca^2+^]_i_ variance in rat neonatal myocytes increases during the action potential–evoked [Ca^2+^]_i_ transient. However, in these cells, thapsigargin decreased [Ca^2+^]_i_ variance by only ∼50%.

[Ca^2+^]_i_ transient variance is a combination of the variance of SR Ca^2+^ release (σ^2^_*SR*_) and Ca^2+^ influx (σ^2^_*influx*_). Thus, in principle, σ^2^_*transient*_ and σ^2^_*influx*_ could be experimentally determined from [Ca^2+^]_i_ transients before and after application of thapsigargin such that σ^2^_*SR*_ = σ^2^_*transient*_ − σ^2^_*influx*_, as was done by Vega et al. (2011). However, a critical condition that this approach must meet is that SR Ca^2+^ release and Ca^2+^ influx are independent, so σ^2^_*influx*_ must not be altered by elimination of SR Ca^2+^ release. Clearly, this is not the case: all the proteins involved in Ca^2+^ influx in these cells (e.g., L-type Ca^2+^ channels and Na^+^/Ca^2+^ exchanger) are modulated by Ca^2+^. A key question is how much of this Ca^2+^ variance comes from the SR versus Ca^2+^ entry.

Previous work has shed light on this issue. Consider rat neonatal myocytes. In these cells, Ca^2+^ influx occurs mostly via L-type Ca^2+^ channels (∼47%) and Na^+^/Ca^2+^ exchanger (∼32%), but T-type Ca^2+^ channels (∼3%) also contribute to Ca^2+^ influx during the action potential (Korhonen et al., 2009). In myocytes isolated from neonatal rats 10 d old or younger, Huang et al. (2006) found that cross talk between SR Ca^2+^ release and the L-type Ca^2+^ current in the form of CICR and SR Ca^2+^ release-induced Ca^2+^-dependent inactivation of L-type Ca^2+^ channels was not detectable. This finding was attributed to a general lack of T-tubules and dyadic structures (where Ca_V_1.2 channels and RYRs coexist in a nanodomain) in these young neonatal rat cardiomyocytes. For the same reason, the impact of SR Ca^2+^ release on Ca^2+^ influx via T-type Ca^2+^ currents in rat neonatal myocytes is likely very small (Cazade et al., 2017).

The functional consequences of SR Ca^2+^ release on Ca^2+^ transport via the Na^+^/Ca^2+^ exchanger during the action potential of neonatal rat cardiomyocytes are more difficult to assess, as this process is largely modulated by global changes in [Ca^2+^]_i_. Even though Ca^2+^ influx represents a large fraction of the Ca^2+^ in the cytosol during the action potential, SR Ca^2+^ release in neonatal rat cardiomyocytes is significant (Korhonen et al., 2009). Thus, although SR Ca^2+^ release is not likely to alter the gating of L- or T-type Ca^2+^ channels in neonatal myocytes from young (≤10-d-old) rats, uncertainties associated with the Na^+^/Ca^2+^ exchanger make it hard to determine if the independence condition is met. Accordingly, the nearly 2.2-fold (∼56%) decrease in [Ca^2+^]_i_ variance seen in rat neonatal myocytes upon elimination of SR Ca^2+^ release should not be considered a quantitatively accurate separation of σ^2^_*SR*_ and σ^2^_*influx*_. Rather, the numbers suggest that, to a first approximation, SR Ca^2+^ release is likely a major driver of [Ca^2+^]_i_ variance during EC coupling in rat neonatal myocytes.

In adult myocytes, Ca_V_1.2 channel gating and SR Ca^2+^ release are not independent, as multiple studies have reported that SR Ca^2+^ release contributes to Ca^2+^-dependent inactivation of Ca_V_1.2 currents in rat (Adachi-Akahane et al., 1996; Sham et al., 1995) and mouse (Masaki et al., 1997) ventricular myocytes. Thus, precise quantitative separation of σ^2^_*transient*_ and σ^2^_*influx*_ cannot be achieved by simply subtracting σ^2^_*influx*_ from σ^2^_*transient*_. That said, because blocking SR Ca^2+^ release reduces σ^2^_*transient*_ by nearly 10-fold in mouse and rabbit adult ventricular myocytes, the conclusion that SR Ca^2+^ release drives most of the noise of the Ca^2+^ transient seems generally correct, as [Ca^2+^]_i_ noise seems small compared with noise when SR Ca^2+^ release is intact.

These studies raise a series of interesting issues and challenges regarding the analysis of noise in cardiac EC coupling. First, what are the precise mechanisms that make SR Ca^2+^ release the largest source of noise associated with the [Ca^2+^]_i_ transient? We do not know the answer to this question, but multiple factors, including variations in sarcolemmal–jSR distance, RYR trafficking, RYR cluster size and location, regional variations in dyadic SR and jSR [Ca^2+^], and stochastic variations in RYR cluster activation, could contribute noise. Indeed, because SR Ca^2+^ amplifies Ca^2+^ influx, it is more likely to be a larger source of noise during EC coupling than sarcolemmal Ca^2+^ movements, at least in part because of the mechanisms discussed above. Other questions include the following: What are the relative contributions of specific events to σ^2^_*influx*_ (e.g., Ca_V_1.2 and Na^+^/Ca^2+^ exchanger trafficking, Ca_V_1.2 current activation and inactivation, and Ca_V_1.2-to-Ca_V_1.2 coupling/function) and σ^2^_*SR*_ (e.g., CICR, RYR clustering, jSR stability, and Ca^2+^ spark amplitude variability)? Does activation of β-adrenergic receptor (βAR) signaling alter σ^2^_*transient*_, σ^2^_*SR*_, and/or σ^2^_*influx*_? Is σ^2^_*transient*_ increased during hypertrophy and heart failure? Finally, what are the mechanisms leading to different [Ca^2+^]_i_ noise values across species and developmental stages?

The development of new experimental and in silico methodologies is needed for accurate, quantitative determination of biological (as opposed to instrument or environment) sources of [Ca^2+^]_i_ noise during EC coupling in ventricular myocytes under a wide range of physiological and pathological conditions. Importantly, these data will be critical for the generation of realistic mathematical models of EC coupling, because, as noted above, most models do not incorporate experimentally determined levels of biological noise.

## Ca_V_1.2 channel trafficking and cluster formation

Recent studies using a combination of superresolution and electrophysiological approaches have revealed important details about the mechanisms that control the organization of Ca_V_1.2 channels in the sarcolemma of ventricular myocytes (Del Villar et al., 2021; Ito et al., 2019; Sato et al., 2019), where Ca_V_1.2 channels, like many other channel proteins, form clusters (Block et al., 1988; De La Mata et al., 2019; Del Villar et al., 2021; Gathercole et al., 2000; Ito et al., 2019; Sato et al., 2019; Yang et al., 2020). Delivery of Ca_V_1.2 channels to the membrane likely occurs via vesicles transported by molecular motors moving along microtubules (Del Villar et al., 2021; Ghosh et al., 2018).

Ca_V_1.2 clusters in the sarcolemma of ventricular myocytes are formed by a stochastic self-assembly process (Sato et al., 2019) such that cluster size depends on the probabilities of Ca_V_1.2 cluster nucleation, growth, and removal. The sizes and densities of Ca_V_1.2 channel clusters reach steady-state levels (Del Villar et al., 2021; Sato et al., 2019), suggesting that plasma membrane expression levels and clustering of Ca_V_1.2 channels are under the control of a feedback mechanism (Rosati et al., 2011). The steady-state Ca_V_1.2 channel number can be viewed as a deterministic set point dictated by the rates of insertion and removal of the channels. This tendency of Ca_V_1.2 channels to achieve a steady-state cluster size and density is an important aspect of Ca^2+^ signaling and EC coupling, as it helps maintain cardiac performance relatively constant under a wide range of physiological conditions. The specific steady-state values would depend on the type of stimulus applied to the myocytes.

An interesting prediction of the Sato et al. (2019) model is that the variation (i.e., noise) associated with any steady-state level in Ca_V_1.2 channel number, which follows a binomial distribution, is influenced by the channel’s membrane dwell time. In this model, an increase in insertion and/or removal rates of channel clusters is associated with an increase in noise in the system, with Ca_V_1.2 cluster numbers fluctuating about the steady state. Accordingly, one can think of the noise introduced by the rapid insertion and removal rates of Ca_V_1.2 channels as a force driving the system away from steady state. However, while the number of channel clusters seems erratic, with no observable period or amplitude, the system fluctuates as it is driven toward steady state via a feedback mechanism but constantly overshoots its destination because of the noise associated with stochastic fluctuations in channel insertion and removal. In principle, this oscillatory behavior could allow for relatively rapid changes in Ca_V_1.2 cluster numbers in the sarcolemma of ventricular myocytes.

Two elegant papers from the Dixon lab (Del Villar et al., 2021; Ito et al., 2019) provided important insights into the mechanisms that regulate Ca_V_1.2 channel trafficking during activation of βAR signaling in ventricular myocytes, showing that Ca_V_1.2 channel insertion increases rapidly (i.e., in 1–5 s) following βAR stimulation and that Ca_V_1.2 channels are often inserted into the sarcolemma as preformed, multichannel clusters. This rapid increase in Ca_V_1.2 channel number seems to result from the fusion of channel-containing reserves of early and recycling endosomes and subsequent transport of endosomes containing Ca_V_1.2 channels by microtubule-associated molecular motors (Del Villar et al., 2021). Microtubules are likely anchored to the sarcolemma by the protein BIN1, which binds Ca_V_1.2 channels and promotes their clustering (De La Mata et al., 2019; Hong et al., 2010).

The work by Ito et al. (2019) and Del Villar et al. (2021) raises many interesting and important questions. For example, is the βAR-induced upward shift in the steady state of sarcolemma Ca_V_1.2 channel numbers associated with an increase in the amplitude of oscillations in channel cluster number and size associated with the new steady state? In addition, does an increase in the variance of Ca_V_1.2 channels, and thus variance of Ca^2+^ influx, contribute to the increase in the probability of arrhythmogenic voltage fluctuations during βAR signaling? Finally, what are the mechanisms that control the new steady-state number during βAR signaling? Future studies should address these important questions.

Beyond its implications for our understanding of the mechanisms involved in controlling cardiac function during βAR signaling, the work by Ito et al. (2019) and Del Villar et al. (2021) also provides insights into cellular processes that lead to the stochastic self-assembly of Ca_V_1.2 channels in ventricular myocytes. Consider these authors’ observation that microtubules are required for Ca_V_1.2 insertion. As shown by Drum et al. (2016), using noncultured adult ventricular myocytes, microtubules are not static cytoskeletal structures, but instead undergo periods of rapid growth, shrinkage, and catastrophe. Importantly, these fluctuations in microtubule length are fundamentally random (Howard and Hyman, 2009). Furthermore, transport along microtubules can occur via a lattice diffusion mechanism (Cooper and Wordeman, 2009; Helenius et al., 2006), also referred to as diffusional motility, which is also random. Thus, as expected, Ca_V_1.2 channel trafficking and cluster formation depends on a plethora of random events. Follow-up studies should investigate whether changes in microtubule and actin dynamics (e.g., growth, shrinkage, and catastrophe) and transport contribute to fluctuations in Ca_V_1.2 channel number in ventricular myocytes.

## Fluctuations in Ca_V_1.2 conductance during diastole and systole

To establish a quantitative framework for variations in the macroscopic Ca_V_1.2 current (*I*_*Ca*_), we begin by noting that *I*_*Ca*_ in a cardiac myocyte is related to the number (*N*) of functional Ca_V_1.2 channels in the surface membrane, the fraction of channels open (*F*_*o*_), and the amplitude of their unitary currents (*i*_*Ca*_). Thus, variations in *I*_*Ca*_ can be produced by stochastic fluctuations in *N*, *F*_*o*_, or *i*_*Ca*_.

Having discussed the mechanisms that regulate Ca_V_1.2 channel insertion and clustering (i.e., *N*) in the sarcolemma of ventricular myocytes, we extend our analysis to a consideration of how *P*_*o*_ and *I*_*Ca*_ are regulated and how this regulation could lead to local and global beat-to-beat variations in Ca^2+^ influx. In the simplest scenario, single Ca_V_1.2 channels gate randomly. This case is not considered just because it is the simplest, but also because the stochastic self-assembly model of Ca_V_1.2 clustering predicts that not all Ca_V_1.2 channels would form multichannel clusters (Sato et al., 2019), a predication with implications for channel gating (see below). Indeed, solitary Ca_V_1.2 channels likely represent a significant fraction of the entire sarcolemmal channel population.

At the maximum diastolic potentials of ventricular myocytes (i.e., −80 to −70 mV), the probability of spontaneous transitions between closed and open states of single Ca_V_1.2 channels is very low, but the probability of transitioning from open to closed is relatively high. As shown by Ca_V_1.2 sparklet studies, Ca_V_1.2 channels can open and allow Ca^2+^ entry even at hyperpolarized potentials (Dixon et al., 2012; Navedo et al., 2005). At present, however, the relative contribution of spontaneous Ca_V_1.2 channel openings to diastolic noise is unclear. Also not known is whether increased openings of Ca_V_1.2 channels at diastolic membrane potentials promote pacemaking in SA node myocytes in addition to increasing basal voltage fluctuations in ventricular myocytes.

Multiple factors could increase variability in Ca^2+^ entry during an action potential. For example, both beat-to-beat fluctuations in the fraction of channels open and the degree of Ca^2+^-dependent inactivation of Ca_V_1.2 channels could contribute to Ca^2+^-influx variability. In addition, variations in the action potential waveform could promote variability in Ca^2+^ entry. Changes in phase 1 and 2 of the action potential are the most likely contributors to beat-to-beat, random fluctuations in Ca^2+^ entry. Indeed, decreasing late openings of Ca_V_1.2 channels during phase 2 of the ventricular action potential decreases arrhythmogenesis (Angelini et al., 2021).

To attain a more complete view of the factors that contribute to Ca^2+^ influx variability, we must also consider Ca^2+^ entry via Ca_V_1.2 channel clusters, as recent studies suggest that the activity of these channels is critically dependent on their spatial organization (reviewed by Dixon et al. [2021]). Clustered Ca_V_1.2 channels physically interact via their C-terminal tails, an interaction that is tightly regulated by local and global [Ca^2+^]_i_. The cascade of events that culminates in the coupling of Ca_V_1.2 channels during an action potential begins with the gating of an individual channel within a cluster. The resulting Ca_V_1.2 sparklet induces the binding of Ca^2+^ to calmodulin in the pre-IQ domain of the channel, which promotes physical interactions between contiguous channels. This increases the activity of adjoined channels, elevating local [Ca^2+^]_i_. As individual channels within a cluster undergo Ca^2+^-dependent inactivation and close, [Ca^2+^]_i_ decreases and coupled channels disassemble. This, in turn, decreases channel opening probability and terminates Ca^2+^ flux. Thus, the overall activity of Ca_V_1.2 channels within a cluster depends on the number of channels that form dimers or higher-order oligomers.

This model suggests that local Ca^2+^ influx via a cluster could fluctuate depending on multiple factors. First, cluster size. Larger clusters are more likely to have more Ca_V_1.2-to-Ca_V_1.2 interactions and hence coupled gating. Such coupled gating has physiological relevance for βAR signaling (Ito et al., 2019), BIN1 function (De La Mata et al., 2019), and Kv2.1 channel activity (O’Dwyer et al., 2020; Vierra et al., 2019), as well as pathological ramifications, as reflected in interactions with mutant Ca_V_1.2 channels in Timothy syndrome (Dixon et al., 2012; Navedo et al., 2010). The situations with mutant channels and βAR signaling are particularly interesting, as interaction of a mutant channel or phosphorylated channel with one or more wild-type or unphosphorylated channels can cause the adjoined channels to function like the channel with the higher *P*_*o*_. Consistent with this, Medvedev et al. (2021) recently reported that cAMP-dependent kinase (PKA)-induced hyperactivation increases cooperative gating of Ca_V_1.2 channels in right ventricular myocytes during hypertrophy. Thus, Ca^2+^ influx may vary depending on the number of mutant or phosphorylated channels within a cluster. Because the composition of a cluster (e.g., number of Timothy syndrome versus wild-type channels) is stochastic, it is a likely source of Ca^2+^-entry noise. The composition of a cluster and the interaction and phosphorylation of a channel with a kinase (e.g., PKA, Ca^2+^/calmodulin kinase II, PKC⍺)—even if bound to an anchoring protein (Carlson et al., 2022; Craske et al., 2005; Smith et al., 2017; Tajada et al., 2017)—are also likely stochastic, and thus are additional sources of Ca^2+^-entry noise.

Second, Ca_V_1.2-to-Ca_V_1.2 proximity and orientation. Channel cluster formation is necessary, but not sufficient, for physical and functional coupling of Ca_V_1.2 channels. Even small, nanometer-scale changes in the distance between channels or orientation with respect to neighboring channels can limit their capacity to interact. Thus, diffusion or removal of one or more channels within a cluster—both random processes—can increase interchannel distances, at least among some of the channels, and hence add variability to coupled gating and hence Ca^2+^ influx.

Third, the degree of Ca_V_1.2–Ca_V_1.2 coupling. Ca_V_1.2 channel coupling is dynamic and varies within the physiological range of [Ca^2+^]_i_ reached in ventricular myocytes during a cardiac cycle (Dixon et al., 2015), exhibiting an apparent *K*_*d*_ of ∼250 nM. This is important because Ca_V_1.2 channel activity remains high for as long as the channels are coupled. Thus, by outlasting the [Ca^2+^]_i_ signal that evoked it, Ca_V_1.2 channel coupling acts as a type of molecular memory that could boost Ca^2+^ influx during repetitive membrane depolarizations. Increased action potential frequency could also boost Ca^2+^ influx by increasing [Ca^2+^]_i_ and thus increase Ca_V_1.2 channel coupling. Accordingly, variations in the number of primed channels could represent a source of Ca^2+^-entry fluctuations.

## jSR formation and stability

To this point, we have discussed how Ca_V_1.2 channels traffic and form clusters, but what about the other side of the dyad—the jSR? The jSR is a complex structure whose architecture, function, and stability reflect the contribution of several proteins (Dixon, 2022; Jones et al., 2018). One of these proteins, junctophilin-2 (JPH2), is anchored to the jSR via its C-terminus and contacts the sarcolemma through lipid-interacting motifs in its N-terminus (Garbino and Wehrens, 2010; Lehnart and Wehrens, 2022; Pritchard et al., 2019). JPH2, which binds to Ca_V_1.2 and RYR2 channels (Feng et al., 2020; Gross et al., 2021; van Oort et al., 2011), is hypothesized to provide a molecular bridge between the jSR and T-tubules. Calsequestrin, a Ca^2+^-binding protein that ensures high concentrations of Ca^2+^ close to RYRs (Beard et al., 2004; Rossi et al., 2008), is anchored to the SR membrane by two proteins, junctin and triadin (Zhang et al., 1997). As a testament to the functional importance of these protein, mice lacking triadin and junctin show changes in jSR architecture and EC coupling (Glover et al., 2002; Zhang et al., 1997).

Until recently, studies investigating the molecular mechanisms that regulate formation of the jSR and dyadic structures were limited to fixed-cell experimental models. A study from our lab by Drum et al. (2020) was the first to image and characterize jSR movement in real time. This study showed that the jSR is dynamic and exhibits several movement modalities, approaching or withdrawing from the sarcolemma and moving laterally to fuse with adjacent jSRs. Indeed, Drum et al. (2020) estimated that “stable” jSR sites had membrane dwell times of >15 min, whereas the residence time for “unstable” jSR membrane sites was as low as 20 s. The overall mean jSR sarcolemmal residence time was ∼4.3 min. Interestingly, the average membrane dwell time of mobile jSRs measured by Drum et al. (2020) was similar to that of Ca_V_1.2 channels in tsA-201 cells and ventricular myocytes (Del Villar et al., 2021; Ghosh et al., 2018; Sato et al., 2019).

In a similar vein, Novotova et al. (2020) independently found using EM that dyads could be broadly classified in two categories—compact and loose—based on the proximity of the jSR to the sarcolemma. We hypothesize that the compact and loose jSRs described by Novotova et al. (2020) correspond to stable and unstable jSRs described by Drum et al. (2020).

Novotova et al. (2020) further suggested that SR Ca^2+^ release by compact dyads in control ventricular myocytes was faster and larger than that in loose dyads. It is likely that the larger SR Ca^2+^ release observed in the compact dyads reported by Novotova et al. (2020) is a manifestation of the strong functional coupling between Ca_V_1.2 and RYRs reported previously by Inoue and Bridge (2003) and Sobie and Ramay (2009). Taken together, the findings of Drum et al. (2020) and Novotova et al. (2020) challenge the traditional view that the jSR is a static structure and that this feature is responsible for maximizing the reproducibility of myocyte responses to an action potential. Instead, it may be that local SR Ca^2+^ release from a similar number of couplons in the myocyte is what perpetuates the reproducibility of the heartbeat under steady-state conditions and that the spatial dwell time of many dyads may be transient.

Several studies provide insight into the potential mechanisms underlying the mobility of the jSR and hence stability of the dyad. JPH2 is necessary for stabilizing the plasmalemma and jSR by providing a structural bridge between membranes; thus, not surprisingly, JPH2 downregulation reduces the number of junctional membranes (van Oort et al., 2011). Two recent studies have further suggested that JPH2 physically binds to Ca_V_1.2 and that this interaction is critical for dyad formation (Feng et al., 2020; Gross et al., 2021; Hennessey et al., 2013; Jiang et al., 2016). Notably, JPH2 also interacts with RYR2s (Beavers et al., 2013; van Oort et al., 2011).

These findings raise an important question: How is the dyad formed? We propose that the first step in the formation of a dyad and couplon is the random insertion of Ca_V_1.2 channels into the sarcolemma. It is likely that nondyadic jSR expressing RYR2 clusters and JPH2 move along randomly distributed microtubules, ultimately reaching the sarcolemma. These jSR–sarcolemmal junctions are more likely to form stable, compact dyads between the sarcolemma and jSR in which large Ca_V_1.2 clusters, RYR2s, and JPH2 are expressed, as they offer more interaction sites through which JHP2 can anchor the jSR to the sarcolemma (Feng et al., 2020; Gross et al., 2021). In this model, the interaction of JPH2 with RYR2s may not simply be functional, it may also be structural. Indeed, assuming a stoichiometric relationship between RYR2 and JPH2, increasing RYR2 would be associated with higher JPH2, and the larger RYR2s would have a higher likelihood of forming dyads. This may be why nonjunctional RYR clusters (presumably jSR terminals) are quite small (Shen et al., 2019). This is important because, as Inoue and Bridge (2003) and Sobie and Ramay (2009) showed, openings of multiple Ca_V_1.2 channels increase the probability of Ca^2+^ spark activation (*P*_*s*_) during the plateau of the ventricular action potential.

Conversely, dyad dissolution, the opposite side of this dynamic relationship, may result from Ca^2+^-dependent proteolysis of JPH2 (Murphy et al., 2013; Wang et al., 2021). Whether dyad dissolution is a necessary step for the removal of Ca_V_1.2 channels from the sarcolemma is unknown, but if true, Ca_V_1.2 channels in dyadic structures would be spared from removal (i.e., left behind) due to structural constraints imposed by the jSR, which could potentially block access for channel removal.

The stochastic assembly model proposed here represents an alternative view of dyad formation compared with the targeted-recruitment models proposed in multiple studies (Jones et al., 2018; Rossi et al., 2008; Zaman et al., 2020). Although the two models achieve the same outcome—high colocalization of Ca_V_1.2 and RYRs at sarcolemmal–jSR junctions—they do so via very different mechanisms. In its simplest formulation, the targeted-recruitment model implies an active process that delivers proteins to very specific sites within the cell. By contrast, the stochastic model relies on a set of random cellular processes, such as Ca_V_1.2 and RYR clustering, microtubule dynamics, and movement of cargo, as well as the binding of JPH2 to Ca_V_1.2 and RYRs, to form dyads. High-resolution time-lapse microscopy of dyad formation in living cells is needed to determine the step-by-step assembly of these critical structures. It is possible that the apparent enrichment of Ca_V_1.2 and RYRs seen at specific sarcolemmal sites in static images may not necessarily reflect targeting but instead indicate channels in dyads that were spared from removal, or were left behind, because the jSR prevented channel removal.

## Variations in SR Ca^2+^ release

During EC coupling, Ca^2+^ entry via Ca_V_1.2 channels stimulates RYR-mediated Ca^2+^ release through a CICR mechanism. At the single-couplon level, SR Ca^2+^ release is regulated by multiple processes, including Ca_V_1.2 channel clustering and coupling, Ca^2+^ entry, sarcolemma–jSR distance, number of RYRs in the Ca^2+^-release unit, RYR phosphorylation, and local SR Ca^2+^ load. Like Ca_V_1.2 channels, RYRs cluster through a stochastic self-assembly mechanism (Baddeley et al., 2009; Soeller et al., 2007). In addition to impacting Ca^2+^ spark amplitude and kinetics (see below), RYR cluster size heterogeneity may potentiate Ca^2+^ waves (Xie et al., 2019).

Variations in SR Ca^2+^-release events may result from variations in RYR clustering (i.e., number of RYRs per cluster and/or channel-to-channel proximity) and local SR Ca^2+^. Accordingly, a critical step in analyzing the mechanisms of SR Ca^2+^ release is analyzing Ca^2+^ spark amplitude distributions. Notably, there are important technical considerations in the analysis of Ca^2+^ spark amplitudes. In their seminal paper, Cheng et al. (1993) reported that Ca^2+^ sparks exhibited a distribution with a clear non-zero mode (or peak). In subsequent papers, however, Cheng et al. (1999) and Izu et al. (1998) showed that Ca^2+^ sparks, as observed in confocal linescan images, should have a monotonically decreasing amplitude distribution, regardless of whether the underlying events are stereotyped, reflecting the stochastic nature of RYR gating. Accordingly, reported Gaussian distributions of manually detected Ca^2+^ sparks are likely the result of subjective detection bias against small-amplitude events. Multiple excellent image-analysis programs that automatically detect Ca^2+^ sparks and thus eliminate manual-detection bias are available (e.g., Tian et al., 2019).

In the first detailed analysis of Ca^2+^ spark amplitude fluctuations during cardiac EC coupling, Bridge et al. (1999) performed a noise analysis of Ca^2+^ sparks at specific sites to obtain an estimate of the number of active RYRs in the dyad during the action potential. They found that each Ca^2+^ spark likely activated >18 RYRs, a value close to that determined using superresolution imaging (∼14 RYRs per cluster; Baddeley et al., 2009).

Local SR Ca^2+^ content is an important regulator of Ca^2+^ spark amplitude and frequency (Cheng et al., 1996; Cheng et al., 1993). Increases in SR Ca^2+^ load increase Ca^2+^ spark amplitude by increasing the open probability of RYRs and by increasing the driving force for Ca^2+^ flux from the SR lumen to the cytosol (Sitsapesan and Williams, 1994). This translates into a very steep relationship between SR Ca^2+^ and Ca^2+^ spark frequency/amplitude in ventricular myocytes (Cheng et al., 1996). Decreases in SR Ca^2+^ load have the opposite effect. An interesting conclusion of a computational study by Stern et al. (2013) is that Ca^2+^ spark termination by local SR depletion is variable, in some cases causing prolonged Ca^2+^ sparks that terminate through stochastic attrition of the underlying RYRs. Thus, the relationship between local SR Ca^2+^ load and Ca^2+^ spark amplitude and kinetics appears to be quite complex.

Regional variations in RYR number and clustering can also alter Ca^2+^ spark amplitude and kinetics. For example, a mathematical model by Sato et al. (2006) suggested that if the number of RYRs is too small, consecutive openings are difficult to maintain, and stochastic attrition terminates the release. By contrast, activation of dyads containing large RYR clusters induces a large release of Ca^2+^ that causes rapid local depletion of Ca^2+^ from the jSR that terminates the release (Xie et al., 2019). Thus, the gating modalities of RYRs may depend on their spatial organization in the jSR. Because RYR clustering and gating are stochastic, they are, in principle, a source of noise and fluctuations during the action potential. Indeed, using superresolution imaging, Kolstad et al. (2018) observed dispersion of RYR clusters in ventricular myocytes isolated from a rat model of postinfarction heart failure. This resulted in more numerous, but smaller, clusters that seemingly produced optically silent Ca^2+^-leak events. RYR cluster dispersion was also associated with decreased Ca^2+^ spark amplitude as well as slower kinetics. Thus, nanoscale RYR reorganization during heart failure augments Ca^2+^ leak and slows Ca^2+^-release kinetics, leading to weakened contraction. Future studies should determine whether RYR dispersion is attributable to increased channel diffusion, removal of RYRs, and/or the replacement of existing dyads with new dyads containing fewer, more dispersed RYRs.

Under steady-state conditions, Ca^2+^ fluxes across the sarcolemma and SR membrane are in balance (Eisner et al., 2017). In an elegant set of papers, Eisner and colleagues (Trafford et al., 2001; Trafford et al., 1997) proposed a feedback model in which macroscopic changes in Ca^2+^ influx and SR Ca^2+^ release trigger a self-regulatory event that restores steady-state [Ca^2+^]_i_ transients in ventricular myocytes upon a disturbance or physiological stimulus. For example, a global increase in Ca^2+^ should increase SR Ca^2+^ release. In the Trafford et al. model, however, this increase would be short-lived because the increase in SR Ca^2+^ release would increase Ca^2+^-dependent inactivation of Ca_V_1.2 channels, decreasing Ca^2+^ influx. This, in turn, would decrease [Ca^2+^]_i_, SR Ca^2+^ load, and SR Ca^2+^ release. Similarly, an increase in SR Ca^2+^ load due to SERCA stimulation would increase SR Ca^2+^ release, which would increase Ca^2+^-dependent inactivation and hence eventually decrease load and thus release.

As reviewed by Eisner et al. (2020), a key element of this model is how diastolic [Ca^2+^]_i_ is regulated in cardiac muscle. In their model, Ca^2+^ entry via Ca_V_1.2 channels is not the only contributor to this process; the Na^+^/Ca^2+^ exchanger and nonselective cation channels could also bring Ca^2+^ into the cell. At present, however, how all Ca^2+^ regulatory pathways contribute to the regulation of diastolic [Ca^2+^]_i_ is incompletely understood.

## The reproducibility of action potentials and the reliability of EC coupling increase as the number of couplons increases

At this point, it would be fair to ask how it is possible to reconcile all the fluctuations in Ca_V_1.2 and RYR clustering and gating, as well as sarcolemma–jSR distance, described above—which occur at the single-molecule and subcellular level—with the high reproducibility of EC coupling in normal cardiac myocytes. The answer to this question is that, while most of the processes we have described are fundamentally stochastic, this stochasticity is often negligible in the macroscopic world because, at steady state, a system with *N* degrees of freedom would have fluctuations that scale to 1/√*N*.

**Figure 8.**
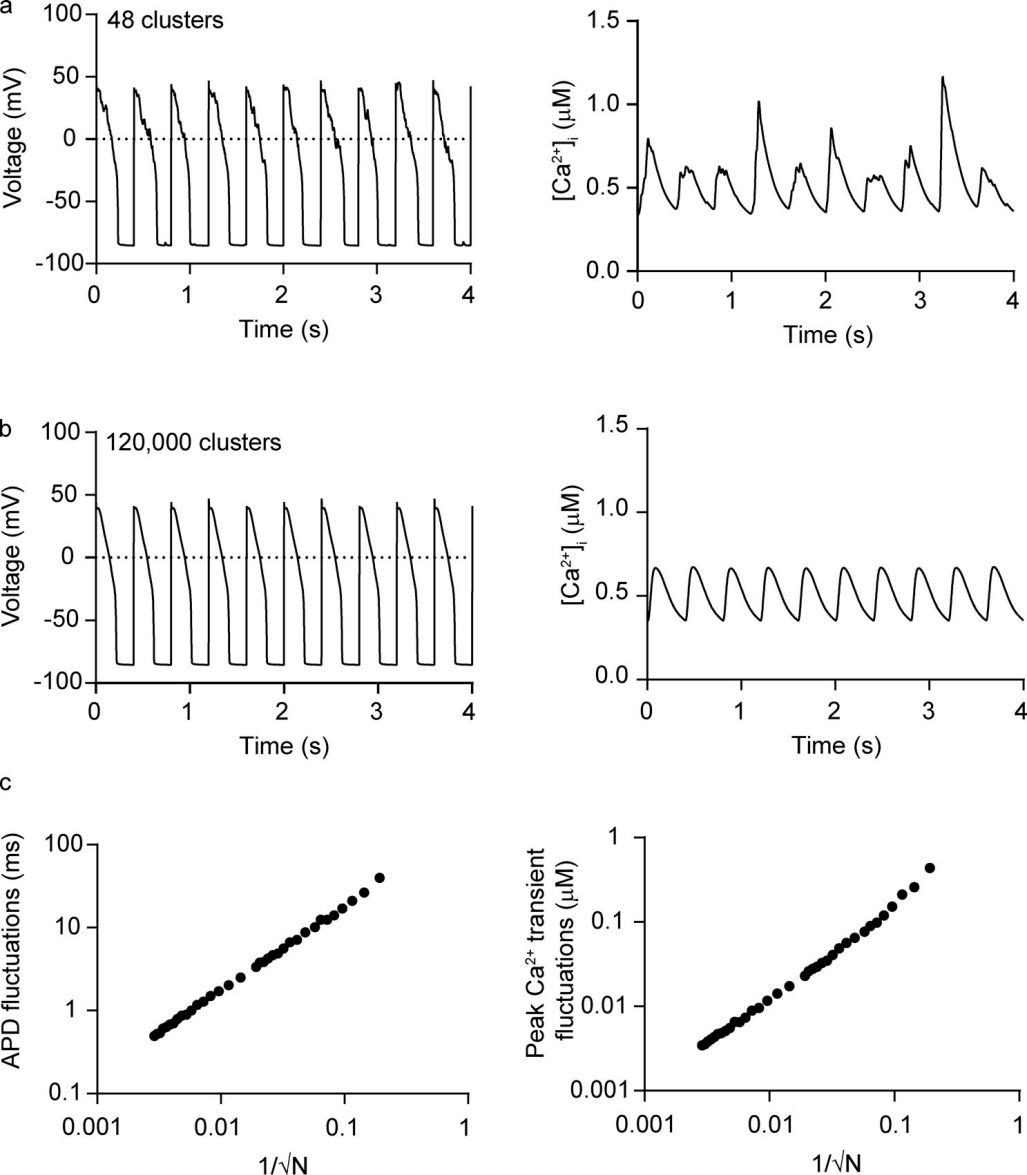
**Increasing the number of SR Ca**^**2+**^**-release units decreases cardiac EC coupling variability. (a and b)** Simulations of action potentials and [Ca^2+^]_i_ transients using a model with a small (a) or large (b) number of SR Ca^2+^-release units. **(c)** Beat-to-beat action potential duration (APD; left) and peak [Ca^2+^]_i_ transient fluctuations (right) versus 1/√*N*, where *N* is the number of SR Ca^2+^-release units.

To illustrate this point quantitatively, we performed simulations using a previously published model of cardiac EC coupling in which we varied the number of SR Ca^2+^-release units (Fig. 8; Sato et al., 2013; Sato et al., 2006; Sato et al., 2021; Sato et al., 2010). Fig. 8, a and b, shows simulated records of action potentials and [Ca^2+^]_i_ (2.5 Hz) in two cells: one with 48 couplons and one with 120,000 couplons. Note that the waveform of the action potential was more heterogeneous in the virtual cell with fewer couplons than in the cell with a relatively large number of couplons. Similarly, Fig. 8, b and c, shows that beat-to-beat [Ca^2+^]_i_ transient fluctuations were larger in the simulations with fewer couplons (a, bottom; SD = 221 nM) than in those with a larger number of couplons (b, bottom; SD = 2 nM).

The results of these simulations suggest that fluctuations in action potential duration and [Ca^2+^]_i_ transients are proportional to 1/√*N*, where *N* is the number of Ca^2+^-release units (Fig. 8, c and d). Importantly, this analysis suggests a potential answer to the question of why SR Ca^2+^ release–associated variance is higher in neonatal than adult ventricular myocytes; namely, that neonatal myocytes have a smaller number of couplons than adult myocytes. Future work should test this intriguing hypothesis.

Notably, the variance of [Ca^2+^]_i_ transients in the modeled cell with 120,000 couplons—a realistic value for a normal adult rabbit ventricular myocyte—was on average ∼74-fold smaller (4.1 nM^2^) than the experimentally determined mean peak [Ca^2+^]_i_ variance during the action potential in such myocytes (i.e., 297.4 ± 44.3 nM^2^). Although instrument and environment sources of noise contribute to experimental [Ca^2+^]_i_ variance, they are not likely the main drivers of the difference between experimental and model levels of noise. There are two reasons for this. Instrument and environment noise should be constant throughout the duration of the experiment, whereas diastolic [Ca^2+^]_i_ variance is low (i.e., 11.2 ± 1.5 nM^2^) and increases during the action potential. Similarly, instrument and environment noise should be insensitive to thapsigargin, yet thapsigargin decreased [Ca^2+^]_i_ variance nearly 10-fold during the rabbit action potential. The large discrepancy between in silico versus experimental noise raises the possibility that biologically driven [Ca^2+^]_i_ noise is underestimated in computational models of EC coupling. It is therefore imperative that future models of EC models incorporate realistic levels of Ca^2+^ variance and examine its physiological implications, as done by the labs of Rodriguez, Clancy, and Smith (Britton et al., 2013; Gemmell et al., 2014; Kernik et al., 2019; Lachaud et al., 2022; Lawson et al., 2018).

## Noise increases cardiac arrhythmogenesis in disease

A large body of work suggests that there are many intraindividual (e.g., regional) and interindividual variations in action potential waveform that manifest as dispersion in different sections of the electrocardiogram (Chou et al., 2007; Hinterseer et al., 2010; Hondeghem et al., 2001; Johnson et al., 2010; Myles et al., 2010; Myles et al., 2008; Ripplinger et al., 2009). This is likely due to variations in the activity and kinetics of ion channels underlying cardiac muscle action potentials. Experimental and computational analyses of this variance are important, as increased action potential variability in ventricles increases the probability of arrhythmias (Zhang et al., 2011).

Particularly relevant to this review, multiple studies have shown that changes in the T-tubule sarcolemma–SR junction likely contribute to [Ca^2+^]_i_ instability during pathology. For example, Zhang et al. (2013) and Wei et al. (2010) found that T-tubules and the SR physically remodel during the development of heart failure. Furthermore, Louch et al. (2013) showed that, in heart failure, dyssynchronous Ca^2+^ transients are a result of T-tubule disorganization, which, in turn, is associated with a decrease in JPH2 expression (Reynolds et al., 2016). Song et al. (2006) found that remodeled T-tubules move away from the Z-lines in heart failure, leading to loss of local control and Ca^2+^ instability. Similarly, Wagner et al. (2012) found T-tubule remodeling as well as early EC uncoupling and SR network fracturing after myocardial infarction. Notably, some T-tubules located away from Z-lines could be newly developed axial tubules, which form couplons. That said, the consistent finding is that a net loss of T-tubules, and hence a decreased number of couplons, is a general feature of ventricular myocytes during the development of hypertrophy and heart failure (recently reviewed by Dibb et al. [2021]).

In general, the loss of T-tubules and consequent loss of dyadic structures have been suggested as a cause for decreased contractility during pathological conditions. However, as suggested by the simulations in Fig. 8, the greater the loss of dyads, the larger the predicted variability in action potentials and [Ca^2+^]_i_ transients, which could be arrhythmogenic. Early and delayed afterdepolarizations and Ca^2+^ alternans—alternating (high-low-high) beat-to-beat variations in the amplitude of [Ca^2+^]_i_ transients—are common sources of arrhythmogenic behavior. Early afterdepolarizations (EADs), which occur when inward currents exceed outward currents during the plateau phase of the action potential, can be caused by reactivation of Ca_V_1.2 channels (Madhvani et al., 2015; Madhvani et al., 2011). The reactivation of these channels is random. Once depolarization starts, it self-amplifies through positive feedback to create a large spike. These EADs can be chaotic (Sato et al., 2009; Tran et al., 2009; Xie et al., 2014), which means the dynamics underlying EADs are extremely unstable. In this case, fluctuations significantly affect the formation of EADs (Sato et al., 2010).

**Figure 9.**
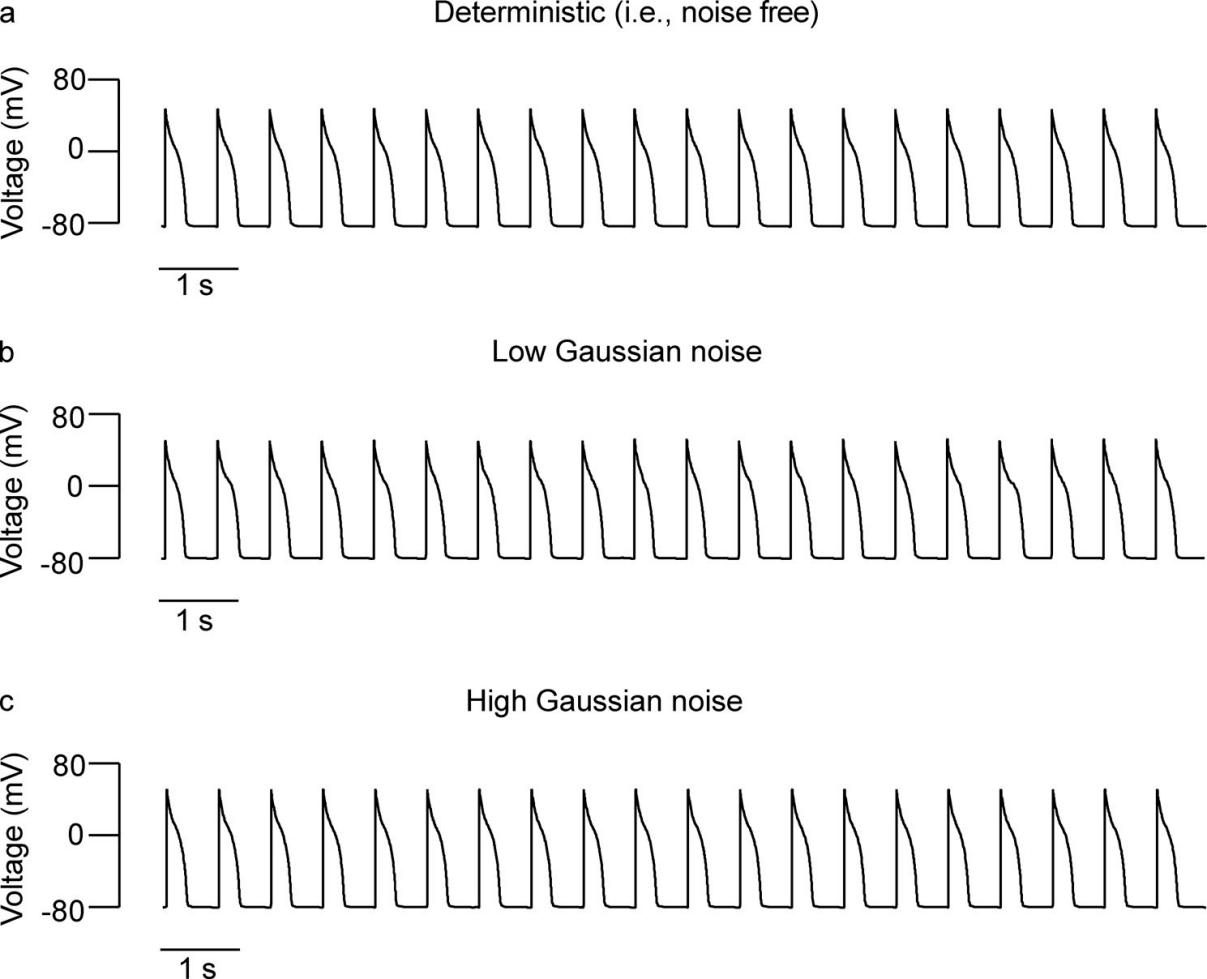
**Noise has a limited impact on arrhythmogenesis in cases where ventricular myocytes have a high repolarization reserve. (a–c)** Action potential simulations using zero (a), low (b), and high (c) Gaussian noise levels.

**Figure 10.**
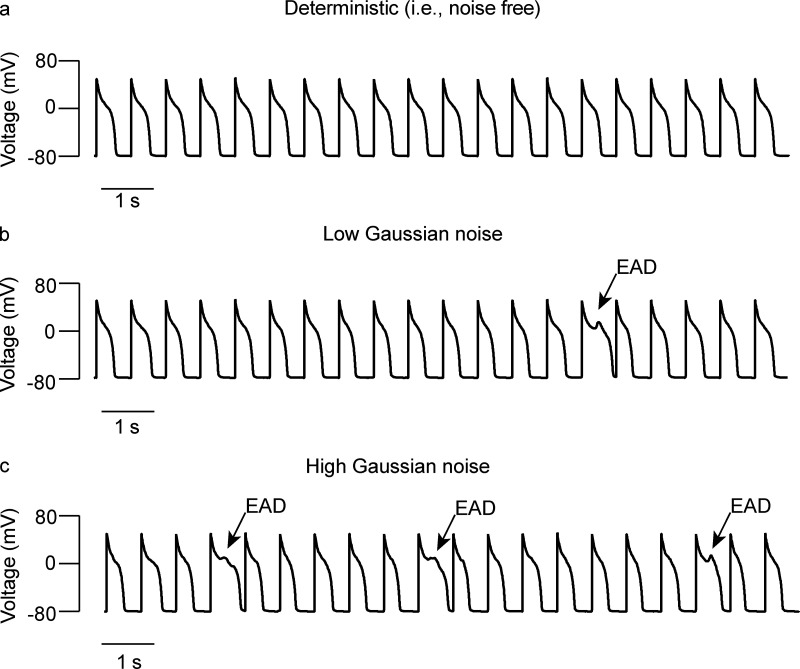
**Noise increases the probability of EADs when repolarization reserve is decreased by 50%. (a–c)** Action potential simulations using zero (a), low (b), and high (c) Gaussian noise levels.

A reduction in voltage-gated K^+^ currents is a hallmark of the electrical remodeling that takes place in ventricular myocytes during the development of pathological hypertrophy and heart failure (Beuckelmann et al., 1993; Nuss et al., 1999; Qin et al., 1996; Rossow et al., 2004). This reduction is typically associated with action potential prolongation and decreased repolarization reserve, which collectively increase the probability of EADs and arrhythmogenesis. Using the model of Sato et al. (2010), we ran simulations with 0, low, and high levels of Gaussian noise in two scenarios: normal conditions (Fig. 9; i.e., normal ionic conductances) and conditions in which the *K*_*s*_ conductance was decreased by 50% (Fig. 10; i.e., decreased repolarization reserve). These simulations showed that, under normal conditions, increasing levels of Gaussian noise do not increase the probability of EADs (Fig. 9, a–c). However, when repolarization reserve is decreased, even low levels of noise increase the probability of EADs (Fig. 10, a–c).

Changes in the spatial domain (such as random variations in intracellular RYR organization), as well as the strength of cell-to-cell coupling, can also be arrhythmogenic. For example, Galice et al. (2018) and Xie et al. (2019) suggested that stochastic variations in RYR cluster size among individual jSR units could increase local SR Ca^2+^ heterogeneity and increase the probability of arrhythmogenic events. At the multicellular level, Lang et al. (2017) showed that heterogeneous cell-to-cell coupling promotes premature ventricular contractions during heart failure.

**Figure 11.**
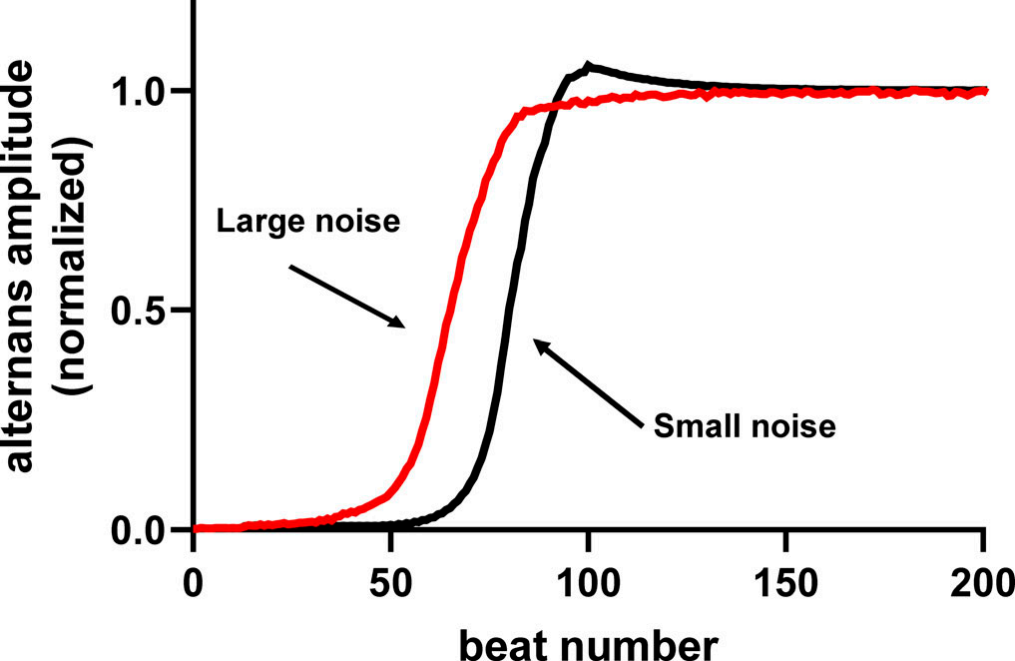
**Noise facilitates development of alternans.** Normalized alternans amplitude (average of 20 simulations) versus beat number. Initial conditions and parameters are the same except the number of SERCA pumps. If the number of SERCA pumps is large (10,000), noise is small (black).

Intrinsic and extrinsic fluctuations can also affect alternans. Voltage fluctuations play a critical role in alternans, especially when these events are driven by unstable Ca^2+^ dynamics (Sato et al., 2006; Shiferaw et al., 2005). The phase of alternans can be either a long-short-long-short pattern or a short-long-short-long pattern. During the development of alternans, the pattern is determined by initial conditions of the cell and fluctuations in action potential duration (Madhvani et al., 2015; Sato et al., 2013). If there are no fluctuations, the pattern is determined only by the initial conditions. However, in cases where there are fluctuations in the action potential waveform, these fluctuations facilitate the development of alternans (Fig. 11). New simulations we performed using the model of Sato et al. (2013) showed the development of alternans (0, no alternans; 1, fully developed alternans) with small and large noise levels, reflecting simulations with a large number (10,000) and small number (100) of SERCA pumps, respectively. Solid lines are averages of 20 simulations. Note that as noise becomes larger, alternans develop earlier. This suggests that Gaussian noise facilitates the development of alternans. Furthermore, in the context of experimentally determined [Ca^2+^]_i_ variance data, the probability of a cell developing arrhythmogenic Ca^2+^ signals my vary depending on its intrinsic level of noise.

## Nonbiological sources of noise and sampling considerations

To this point, we have discussed how stochastic events contribute to variations in Ca^2+^ signaling in cardiac myocytes. We have also considered the importance of experimentally determining the source, magnitude, and time course of these variations and using them to inform simulations with computational models. However, the variance in imaging and electrophysiological recordings is not just attributable to stochastic variations in biological processes; it also includes environmental noise (e.g., room lighting), electronic noise (e.g., shot noise), and even analytically introduced noise (e.g., Ca^2+^ fluorescence signal calibration). To accurately quantify biological noise, one must therefore eliminate, minimize, or account for these external sources of noise. Although a detailed analysis of nonbiological sources of noise and how they are treated is beyond the scope of this review, we include a brief discussion of this topic so that readers are aware of it.

Any imaging recording is susceptible to contamination with photons produced by environmental light that enters the optical path and reaches the system’s photon detectors. Eliminating this noise simply involves turning off unnecessary lighting, using appropriate filters, and/or physically shielding the microscope from these sources of photons.

Careful selection of the fluorescent Ca^2+^ indicator is critical for any imaging experiment and requires consideration of multiple factors, including brightness, dynamic range, and apparent dissociation constant. Converting fluorescence values or ratios to concentration units is also important, not just for mechanistic reasons but also to accommodate the nonlinear relationship between Ca^2+^ concentration and fluorescence. In cases in which Ca^2+^ levels reach the flatter section of the sigmoidal Ca^2+^–fluorescence relationship, where small changes in fluorescence can translate to large changes in Ca^2+^ and hence greater [Ca^2+^]_i_ variance, this conversion process is an often unappreciated source of variation that contributes Ca^2+^ noise. Furthermore, the magnitude of the noise recorded with a detector such as a PMT (i.e., shot noise) increases with light intensity, adding noise to any recorded [Ca^2+^]_i_. While this source of noise could be a minor contributor to the overall level of [Ca^2+^]_i_ variance in images, it must be taken into consideration during analysis.

Fluorescence microscopes and patch-clamp amplifiers are susceptible to other types of electronic noise produced by its components, such as shot noise, dielectric noise, and operational amplifier noise. Electrical noise from external sources can be almost completely eliminated in a well-designed system but can become the dominant source of noise if proper precautions are not taken. The most familiar form of electrical interference is line-frequency pickup (50 or 60 Hz and harmonics) from power supplies and fluorescent lights, among other sources. Well-designed instruments will not introduce significant amounts of interference from their internal power supplies. However, a typical laboratory environment is full of potential sources of interference from sources external to the electronic instrumentation involved in a particular measurement. In addition to line-frequency pickup, other potential sources of interference include nearby motors, elevators, radio and television stations, and video monitors of the researchers’ computers, which produce an annoying timing signal at frequencies of ≥16 kHz. High-impedance measurements, such as patch-clamp and intracellular microelectrode recordings, are particularly sensitive to such external interference. In most cases, such noise sources can be controlled by careful grounding, shielding, and filtering.

A final consideration is embodied by Nyquist’s theorem, also known as the sampling theorem, which states that a periodic signal must be sampled at more than twice the highest frequency component of the signal. In imaging, the recommended sampling rate is at least 2.3 times the highest frequency. For example, for a ventricular myocyte with a [Ca^2+^]_i_ transient that peaks 50 ms after activation, the cell needs to be imaged with a minimum time resolution of 22 ms (i.e., 50 ms/2.3), or 45 frames or linescans per second, to accurately capture the amplitude of the transient. Meeting Nyquist’s criteria is also important in capturing images with the highest spatial resolution possible with a given microscope configuration, keeping in mind that each pixel is at least 2.3 times smaller than the calculated resolution of the objective. Failure to meet Nyquist’s criteria leads to low spatial and temporal resolution due to undersampling and hence filtering of the signal, which could lead to distortion of the signal’s waveform, including its variance (i.e., noise).

## Summary and future directions

In this review, we have provided an overview of key processes involved in cardiac pacemaking and EC coupling and how their stochasticity contributes to increased variability, but paradoxically is also key to increased periodicity and reproducibility.

Noise has traditionally been seen as random fluctuations that invariably blur events, decreasing detection, and thus decreasing reproducibility. In this view, optimizing function requires minimizing noise. In other words, the relatively high performance of biological systems—in our case the heart—occurs despite noise. We posit that the binary view of noise and signal as two opposing properties is not just limiting, but also counterproductive. This mindset, and our definition of noise and signals, at least in cardiac physiology, must change.

As a start in this direction, we offer two examples in which noise could increase cardiac performance. The first example is from ventricular myocytes. The fluctuations in EC coupling in these cells are associated with stochastic events discussed above, such as ion channel gating, microtubule dynamics, protein clustering, and Ca^2+^ diffusion. These events shape subcellular and cellular responses of ventricular myocytes to physiological stimuli. The second example of how cell-to-cell regional variability in excitability and noise could increase cardiac performance comes from pacemaking by the SA node. A stochastic resonance model of pacemaking (Clancy and Santana, 2020; Grainger et al., 2021), if validated, may show how regional variations in the vasculature and blood flow help determine electrical signaling modalities and thus create noise-producing zones that increase the periodicity of the cardiac cycle. SA node cells would be susceptible to tissue sources of electrical and Ca^2+^ signaling variability (e.g., vascularization) but also to inter- and intracellular sources (e.g., ion channel gating, microtubule dynamics, clustering, etc.).

The ability of highly heterogeneous cellular networks to generate highly periodic signals and respond to physiological stimuli is not unique to the heart, as exemplified by seminal work by Dr. Eve Marder and her colleagues on the mechanisms governing excitability in the crustacean stomatogastric ganglion (see recent review by Goaillard and Marder [2021]). These researchers elegantly demonstrated how networks composed of cells with diverse electrical signaling properties, such as the SA node, can produce highly periodic firing patterns. For example, the strength of the same synapse across animals, as well as the conductance of many membrane currents, can vary by as much as two- to sixfold and still lead to similar stomatogastric ganglion circuit dynamics (Goaillard et al., 2009). Modeling studies have demonstrated that the system is relatively insensitive to variation (noise or heterogeneity) in certain combinations of parameters (Gutierrez and Marder, 2013; O’Leary et al., 2013; O’Leary et al., 2014; Prinz et al., 2004; Tang et al., 2010)—work that has led to the formulation of “good-enough” models of electrical activity in multiple neuronal circuits. However, with some other combination of electrophysiological parameters, the system is more sensitive to variation and therefore more adaptable. Building on these concepts, a recent study by Rees et al. (2018) created a good-enough model of cardiac electrophysiology that predicts multiple variations in ion channel and transporter conductances that generate a normal [Ca^2+^]_i_ transient without restrictions in the action potential. Notably, the model suggests the existence of a feedback mechanism that sets the balance between Ca^2+^ and other conductances to determine the [Ca^2+^]_i_ transient.

Critical information can be generated by applying a similar conceptual framework combining in silico and experimental approaches to the study of stochastic variability in time and space domains, as was done in recent studies (Britton et al., 2013; Gemmell et al., 2014; Lachaud et al., 2022; Lawson et al., 2018). For example, one benefit of combining in silico studies with experimental studies is that it allows for quantitative investigation of parameters critical to the success and robustness of SA node signal generation. Such parameters include the necessary noise-to-subthreshold cell ratios and their influence, as well as intercellular coupling, the source–sink relationship, and the proximity to atrial tissue.

New tools and computational approaches for predicting modulatory effects of peripheral nerve activity are also badly needed for developing new strategies for optimizing heart rate and treating heart rate disorders. The key will be to integrate data from subcellular, cellular, and SA node scales to predict the effect of efferent stimulation of sympathetic and parasympathetic branches of the autonomic nervous system on the cardiac SA node. New representations of sympathetic and parasympathetic branches of the autonomic nervous system and their synapses onto the cardiac pacemaker are under development and will allow connection to the resultant receptor-mediated signaling pathways. Full realization of these developments will allow robust prediction of the effects of autonomic nervous system input on pacemaking and how its coupling drives activity in contractile cells and cellular- and tissue-level responses in electrophysiology.

Computational modeling and simulation might also be extended to allow for quantitative investigation of the electrophysiological and Ca^2+^ signatures of individual cells. It is well known that in vitro approaches and small animal models are not always accurate representations of the human physiological environment. In particular, measurements in cell expression systems and animal models cannot faithfully recapitulate the patient-to-patient variability that often underlies adverse drug/nervous system effects. In principle, experiments might inform a collection of parameters for constructing individual-specific cardiac ion channel models and Ca^2+^-handling parameters, and then, by combining them, develop a patient-specific digital representation. Populations for control (i.e., disease-free) and genetic disease groups linked to inherited arrhythmia could then be used to test the conditions under which stochastic resonance fails.

In an evolutionary context, the variance associated with action potential waveform, subthreshold voltage fluctuations, and Ca^2+^ signaling may offer a selective advantage by allowing for rapid change and adaptation to changing physiological needs. For instance, relatively fast trafficking events that insert and remove Ca_V_1.2 channels into the sarcolemma of ventricular myocytes might decrease the channels’ membrane dwell time but could also facilitate the rapid delivery (i.e., within seconds) of Ca_V_1.2 channels during βAR signaling. The argument here is that if trafficking were a very slow process, it could take a long time to tune cardiac performance through changes in channel numbers. As shown by Dixon and colleagues (Del Villar et al., 2021; Ito et al., 2019), this is critical for the fight-or-flight response. Viewed from this perspective, noise is not an evolutionary accident, but instead provides a way for stochastic events to enable cells to tune their function and quickly adapt to changing physiological demands.

As highlighted above, this stochasticity, while large at the subcellular level, is usually negligible at the whole-cell level because of spatial-temporal summation of individual events within cells. That said, optimal levels of noise can, at least in principle, increase pacemaking at the multicellular level and enhance EC coupling performance at the cellular level.

Although there is a growing body of work demonstrating the massively dynamic nature of cellular processes in cardiac cells and regions, the significance of random fluctuations in electrical and Ca^2+^ signaling at these two organizational levels is not fully realized. Achieving this will require the development of a combination of experimental and in silico methodologies for accurately determining the amplitude and sources of [Ca^2+^]_i_ variance during the action potential. These values can then be fed into mathematical models to generate more realistic simulations of the action potential and [Ca^2+^]_i_.

Additional questions that should be addressed using these experimental and in silico approaches in future studies include the following: What is the noise–performance relationship of the SA node and ventricular myocytes? Does the magnitude and optimal level of noise vary throughout the heart? How do regional variations in action potential waveform and βAR signaling contribute to noise heterogeneity? Is that changed by pathology, and if so, how? And how does loss of T-tubules and dyads alter the reproducibility of EC coupling in living myocytes? Addressing these questions will provide novel insights into the mechanisms that regulate EC coupling but will also require a shift in conceptual and experimental paradigms.
